# AI‐Designed Cyclic Peptides Enable Controllable Modulation of the CD28 Immune Checkpoint

**DOI:** 10.1002/advs.75892

**Published:** 2026-05-30

**Authors:** Katarzyna Kuncewicz, Saurabh Upadhyay, Renjie Zhu, Hongliang Duan, Moustafa T. Gabr

**Affiliations:** ^1^ Department of Radiology Molecular Imaging Innovations Institute (MI3) Weill Cornell Medicine New York New York USA; ^2^ Department of Biomedical Chemistry Faculty of Chemistry University of Gdansk Gdansk Poland; ^3^ Faculty of Applied Sciences Macao Polytechnic University Macao China

**Keywords:** CD28, cytokine, immune checkpoint, immune system, immunotherapy, proinflammatory cytokine

## Abstract

Immune checkpoint therapies have transformed immunotherapy but remain dominated by biologic agents characterized by prolonged receptor occupancy and limited pharmacologic controllability. Although multiple classes of immunomodulatory therapeutics exist, synthetic modalities capable of directly targeting extracellular immune checkpoint protein–protein interaction interfaces while enabling controllable immune modulation remain comparatively underexplored. Here, we report an AI‐guided strategy for discovering cyclic peptide antagonists of the costimulatory receptor CD28. The lead peptide, CIP‐3, binds the CD28 extracellular domain with nanomolar affinity and disrupts CD28‐ligand interactions. In primary human immune systems, CIP‐3 suppresses CD28‐dependent T‐cell activation without intrinsic agonist activity and exhibits rapid pharmacologic reversibility, enabling exposure‐dependent control of immune signaling. In a T‐cell transfer model of chronic colitis, CIP‐3 confers dose‐dependent therapeutic efficacy and reduces systemic inflammatory cytokines. CIP‐3 also suppresses cytokine production across independent healthy donors and patient‐derived PBMCs from individuals with ulcerative colitis with efficacy comparable to a benchmark anti‐CD28 biologic. Together, these findings demonstrate the potential of AI‐designed cyclic peptides as a controllable synthetic modality for immune checkpoint modulation.

## Graphical abstract

AI‐designed cyclic peptides enable controllable modulation of the CD28 immune checkpoint through reversible disruption of CD28‐CD80/CD86 interactions. The lead peptide, CIP‐3, suppresses T‐cell activation without intrinsic agonist activity, demonstrates dose‐dependent efficacy in a murine colitis model, and attenuates inflammatory cytokine production in primary human immune cells from healthy donors and ulcerative colitis patients.

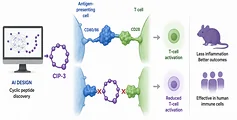

## 1 Introduction

Immune checkpoint pathways govern the magnitude, quality, and duration of adaptive immune responses and have emerged as central therapeutic targets across oncology, autoimmune disease, and transplantation medicine [1, 2, 3]. Therapeutic blockade of inhibitory checkpoints such as PD‐1 and CTLA‐4 has transformed clinical practice and validated immune modulation as a powerful therapeutic strategy [4, 5, 6]. Increasing attention has now turned toward costimulatory pathways that directly regulate T‐cell activation thresholds and effector function [7]. Among these, the receptor CD28 plays a central role in amplifying T‐cell receptor signaling through interactions with its ligands CD80 and CD86 on antigen‐presenting cells, promoting cytokine production, proliferation, survival, and metabolic reprogramming of T lymphocytes through downstream signaling pathways including PI3K/AKT and NF‐κB activation [8, 9, 10]. Dysregulated CD28 signaling contributes to inflammatory and autoimmune diseases, including rheumatoid arthritis and inflammatory bowel disease (IBD), motivating efforts to therapeutically modulate this pathway [11, 12, 13].

To date, immune checkpoint modulation has been dominated by biologic agents, including monoclonal antibodies and receptor fusion proteins such as abatacept and belatacept, which effectively engage extended protein‐protein interaction surfaces [14, 15, 16]. Despite their success, biologics present intrinsic limitations including prolonged receptor occupancy, limited pharmacologic controllability, immunogenicity risk, and complex manufacturing requirements [17, 18, 19]. Moreover, modulation of costimulatory receptors such as CD28 carries unique safety considerations, as excessive receptor activation can trigger severe cytokine release syndromes [20]. These limitations highlight the need for therapeutic strategies capable of precisely tuning immune signaling while maintaining high specificity for receptor interfaces.

Developing synthetic modulators of immune checkpoint receptors remains challenging because their extracellular domains typically present shallow, solvent‐exposed protein‐protein interaction surfaces that lack the deep binding pockets commonly exploited by small molecule drugs [21, 22]. Consequently, most successful therapeutic approaches rely on large biologic agents capable of engaging extended receptor surfaces, leaving a substantial modality gap between conventional small molecules and antibodies [23].

Cyclic peptides represent an emerging molecular class capable of bridging this gap. Their conformationally constrained architectures enable recognition of extended protein surfaces with greater specificity than small molecules while retaining synthetic accessibility, modular optimization potential, and tunable pharmacologic properties distinct from biologics [24, 25, 26, 27]. Several cyclic peptide therapeutics, including cyclosporine A, vancomycin, and octreotide, have demonstrated the clinical utility of conformationally constrained peptide scaffolds across diverse therapeutic areas [24, 25, 26]. Cyclic scaffolds have demonstrated the ability to engage challenging protein‐protein interfaces across diverse biological systems, including intracellular signaling proteins and extracellular receptors [28, 29]. However, systematic strategies for discovering cyclic peptide modulators of immune checkpoints remain limited, in part because efficient exploration of peptide sequence and conformational space poses significant design challenges.

Experimental screening techniques serve as the cornerstone for identifying active cyclic peptide candidates, with high‐throughput methods such as phage display, mRNA display, and DNA‐encoded libraries (DELs) demonstrating significant value in this field. However, despite their large screening capacity, these approaches are inherently time‐consuming, resource‐intensive, and often associated with low overall success rates [30, 31].

Recent advances in AI‐guided molecular design provide new opportunities to address these limitations. Deep learning–based models, such as RFdiffusion, enable precise, structure‐based de novo design of peptide ligands, significantly improving both efficiency and success rates [32, 33]. By shifting from trial‐and‐error screening to an AI‐driven rational design paradigm, these approaches reduce stochasticity and substantially shorten development timelines—from approximately one year in conventional workflows to only a few months. Moreover, machine‐learning approaches integrating structural prediction, sequence optimization, and interaction modeling are increasingly enabling the rational discovery of ligands targeting complex protein interfaces previously considered undruggable [34, 35, 36, 37]. Applying such strategies to immune checkpoint receptors could enable the development of synthetic modulators with improved pharmacologic controllability.

Here, we sought to establish cyclic peptides as a controllable synthetic modality for targeting immune checkpoint receptors. Using an AI‐guided discovery strategy, we identified cyclic peptide antagonists targeting the CD28 immune checkpoint and demonstrated that these molecules enable tunable modulation of T‐cell costimulatory signaling. AI‐designed cyclic peptides achieve nanomolar binding to CD28, disrupt receptor‐ligand interactions, and suppress T‐cell activation across multiple primary human immune cell systems. Importantly, peptide‐mediated antagonism exhibits reversible pharmacology and absence of intrinsic agonist activity, distinguishing it from antibody‐based approaches. Cross‐reactivity with murine CD28 and pharmacokinetic (PK) characterization enabled in vivo evaluation, where cyclic peptide treatment produced dose‐dependent therapeutic efficacy in a T‐cell transfer model of chronic colitis. Together, these findings establish cyclic peptides as a synthetic modality capable of controllable immune checkpoint modulation and provide a framework for next‐generation immunotherapies targeting costimulatory receptors.

## 2 Results

### 2.1 AI‐Guided Discovery Identifies Cyclic Peptide Ligands Targeting the CD28 Extracellular Interface

**FIGURE 1:**
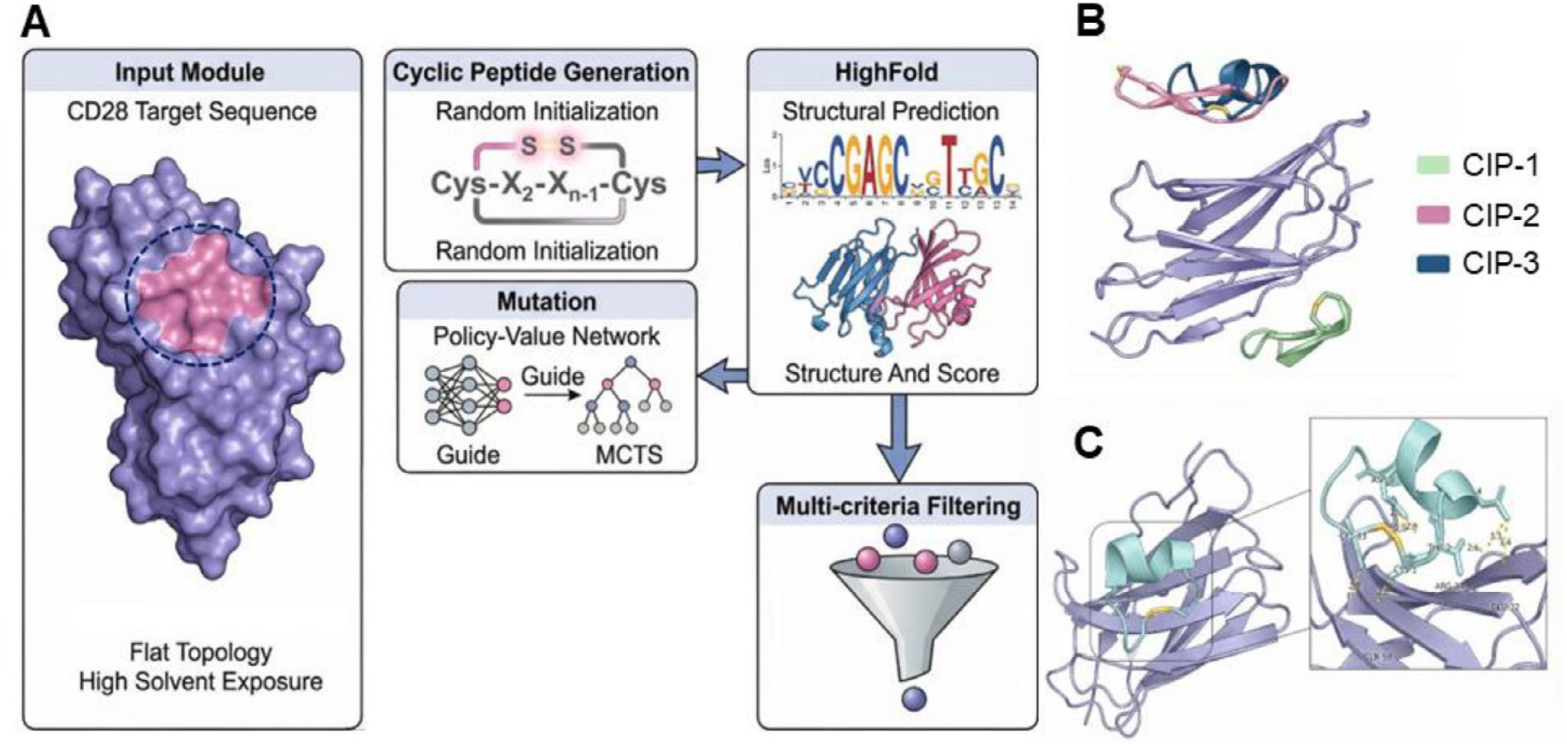
AI‐guided design and structural characterization of cyclic immune peptides targeting CD28. (A) Computational workflow for cyclic peptide discovery. A solvent‐exposed, topologically flat region of CD28 was selected as the target interface and used to guide de novo cyclic peptide generation with a disulfide‐constrained scaffold (Cys‐X_2_‐X_n‐1_‐Cys). Candidate sequences were iteratively optimized using a policy‐value network with Monte Carlo tree search (MCTS) and evaluated by HighFold structural prediction. Multi‐criteria filtering was applied. (B) Structural comparison of the designed cyclic peptides (CIP‐1, CIP‐2, and CIP‐3) bound to CD28. The peptides occupy the targeted interface region identified in panel A. (C) Predicted binding mode of the lead peptide (CIP‐3) in complex with CD28, with inset highlighting key intermolecular interactions and hydrogen bonds stabilizing the interface.

To enable synthetic modulation of the CD28 costimulatory receptor, we developed an AI‐guided discovery framework integrating reinforcement learning‐based sequence optimization with structure prediction to generate cyclic peptides predicted to engage the extracellular domain of CD28 (Figure 1A). CD28 presents a relatively shallow and solvent‐exposed protein‐protein interaction surface, a structural topology that has historically limited the applicability of conventional small‐molecule approaches. In contrast, short peptides can present extended contact surfaces that more closely approximate native ligand geometries, rendering them well suited for targeting interfaces governed by geometry‐dependent extracellular recognition rather than enzymatic activity. Disulfide‐constrained cyclic peptides combine compact size with conformational preorganization, enabling stable presentation of interaction motifs across flat or discontinuous protein surfaces while reducing entropic penalties upon binding and improving resistance to proteolytic degradation.

We therefore prioritized conformationally constrained macrocyclic scaffolds capable of achieving high surface complementarity to the CD28 interface. Using the Highplay platform [38], which integrates Monte Carlo Tree Search‐based reinforcement learning with the HighFold structure prediction model [39], candidate cyclic peptide sequences were generated directly from the CD28 amino‐acid sequence and ranked using predicted interaction energies, interface hydrogen‐bonding potential, and structural confidence metrics derived from ensemble modeling. Filtering criteria included high predicted interface accuracy (i_pLDDT > 0.85) [40, 41] together with favorable intermolecular interaction features, enabling efficient exploration of chemically tractable binders targeting a structurally challenging receptor surface, as i_pLDDT serves as a proxy for interface reliability, while hydrogen bond metrics capture interaction strength and specificity, jointly facilitating the identification of potential high‐affinity binders.

Three disulfide‐constrained cyclic peptides were selected for experimental evaluation. We refer to these molecules as cyclic immune peptides (CIPs), reflecting both their cyclic architecture and intended immune‐modulatory function. Structural modeling predicted that the peptides occupy regions proximal to the CD28 ligand‐binding interface responsible for CD80/CD86 engagement, consistent with a mechanism involving disruption of receptor‐ligand interactions proximal to the CD28 ligand‐binding interface (Figure 1B,C and Figures S1 and S2 for additional models).

Before experimental evaluation, we also conducted ablation studies and benchmarking experiments on the Highplay‐HighFold model to further validate its performance and show the contribution of each key component.

To validate the influence of reward signals on the quality of generated cyclic peptides, we performed ablation experiments targeting CD28. Candidate sequences were designed with lengths ranging from 10 to 14 residues and cyclized via an N─ and C─terminal disulfide bond. We evaluated model performance when using pLDDT, ipTM, and dSASA as the reinforcement learning reward signal, respectively.

As shown in Table 1, using pLDDT as the reward signal resulted in the best search efficiency and sequence quality. In particular, the pLDDT group outperformed the ipTM and dSASA groups in key metrics, including interface model confidence (i_pLDDT > 0.85) and interface hydrogen bond interactions (i_Hbonds > 3). Although the dSASA group performed slightly better in its own area parameter, it showed poor performance in model confidence (only 0.73% for i_pLDDT) and shape complementarity. Based on a comprehensive evaluation of all metrics, pLDDT can more effectively guide the HighPlay platform to select stable cyclic peptides with favorable interfacial interactions from the vast chemical space.

**TABLE 1:** HighPlay ablation experiments.

Reward	i_plddt (>0.85)	dSASA (>1000)	Shape complementarity (>0.7)	Hbonds (>8)	i_Hbonds (>3)
pLDDT	8.15%	57.19%	23.8%	17.25%	59.9%
ipTM	4.99%	50.8%	23.8%	13.34%	41.29%
dSASA	0.73%	68.55%	14.94%	14.83%	42.7%

To validate the applicability and superiority of HighFold in this work, we performed a comprehensive validation on independent cyclic peptide‐protein complex datasets rigorously screened from the PDB database. These test samples cover diverse structural characteristics, with peptide ligands ranging from 6 to 34 residues in length, and encompassing multiple secondary structural motifs such as β‐sheets and random coils.

**FIGURE 2:**
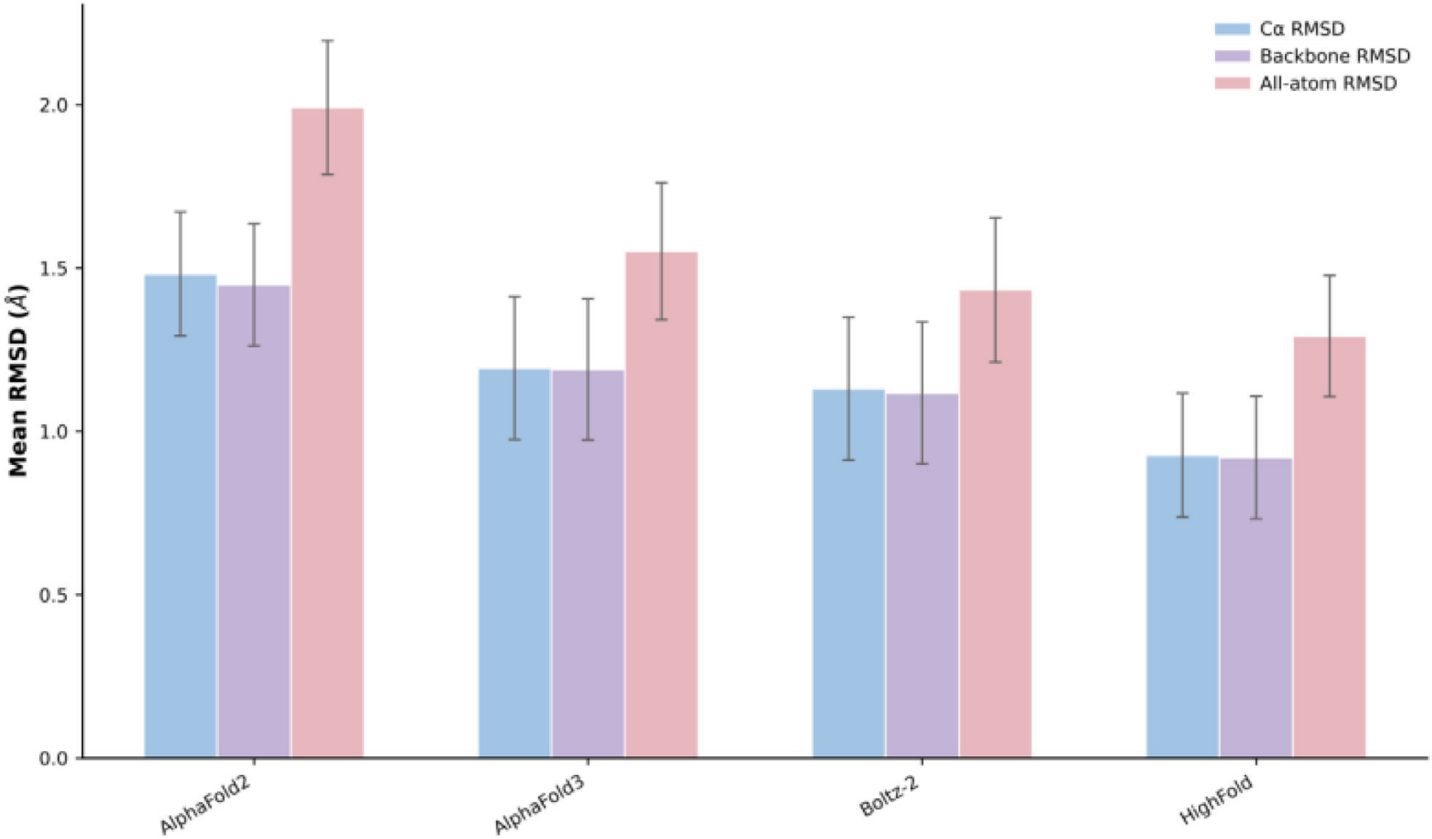
Comparison of RMSD values across different prediction models.

As summarized in the comparison figure (Figure 2), HighFold achieves substantially higher prediction accuracy and stability for complex cyclic peptide–protein complexes compared with other models. Both backbone and all‐atom RMSD values of HighFold remain at a low level (below 1.5 Å). Its robust and consistent performance across varied peptide lengths and secondary structure motifs demonstrates the model's exceptional capability in accurately capturing cyclic conformations and side‐chain geometric features. Such high‐precision structural prediction provides a solid and reliable foundation for subsequent in‐depth analysis of interfacial interactions between cyclic peptides and target proteins.

### 2.2 Cyclic Peptide CIP‐3 Binds CD28 With Nanomolar Affinity

Binding interactions between the designed cyclic peptides and the human CD28 extracellular domain (ECD) were quantified using microscale thermophoresis (MST) under solution‐phase conditions. This approach enables sensitive detection of molecular interactions without immobilization, an important consideration for targets such as CD28 that present relatively shallow, protein‐protein interaction‐driven binding surfaces.

**FIGURE 3:**
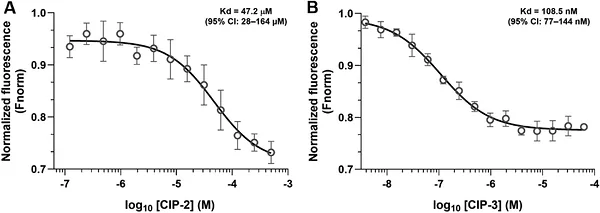
Microscale thermophoresis analysis of cyclic peptide binding to CD28. Binding of disulfide‐constrained cyclic peptides to the human CD28 extracellular domain (ECD) was quantified by microscale thermophoresis (MST) using intrinsic fluorescence detection (spectral shift mode; Monolith X). Normalized fluorescence (Fnorm, 670/650 nm) is plotted as a function of peptide concentration. (A) CIP‐2 exhibits concentration‐dependent binding consistent with a moderate‐affinity interaction. (B) CIP‐3 displays a well‐defined sigmoidal binding curve indicative of higher‐affinity engagement with CD28. Data points represent mean ± SD from three independent measurements. Binding curves were fitted using nonlinear regression, assuming a 1:1 binding model to estimate equilibrium dissociation constants (Kd).

Among the three candidates, CIP‐1 did not exhibit detectable binding across the tested concentration range. CIP‐2 displayed a weak‐to‐moderate concentration‐dependent interaction with an apparent Kd of 47.16 µm (Figure 3A), although the broad 95% confidence interval (28–164 µm) indicates lower precision consistent with weak binding near the assay sensitivity range. In contrast, CIP‐3 demonstrated substantially stronger engagement, yielding a well‐defined sigmoidal binding curve with a Kd value of approximately 108 nm, 95% CI: 77–144 nm (Figure 3B).

Isothermal titration calorimetry (ITC) was used to characterize the interaction between CD28 and CIP3 under near‐physiological conditions. The thermogram (Figure S6) displayed well‐defined exothermic peaks that decreased progressively with successive injections, consistent with a saturable binding process. Integration of the heat signals yielded a smooth binding isotherm with a clear transition and plateau, indicative of a specific interaction. Fitting of the data to a single‐site binding model resulted in a dissociation constant (Kd) of 8.01 × 10^−^^8^ m (∼80 nm) with a stoichiometry (n) of 1, consistent with a one‐to‐one binding interaction. The interaction is associated with a negative enthalpy change (ΔH = −1210 kJ·mol^−^^1^) and a negative entropy change (ΔS = −3923 J·mol^−^^1^·K^−^^1^), reflecting the overall thermodynamic profile of the binding event.

The binding isotherm exhibits a well‐defined saturation profile, supporting robust fitting and consistent estimation of thermodynamic parameters. Collectively, these data demonstrate a specific, high‐affinity interaction between CD28 and CIP3.

**FIGURE 4:**
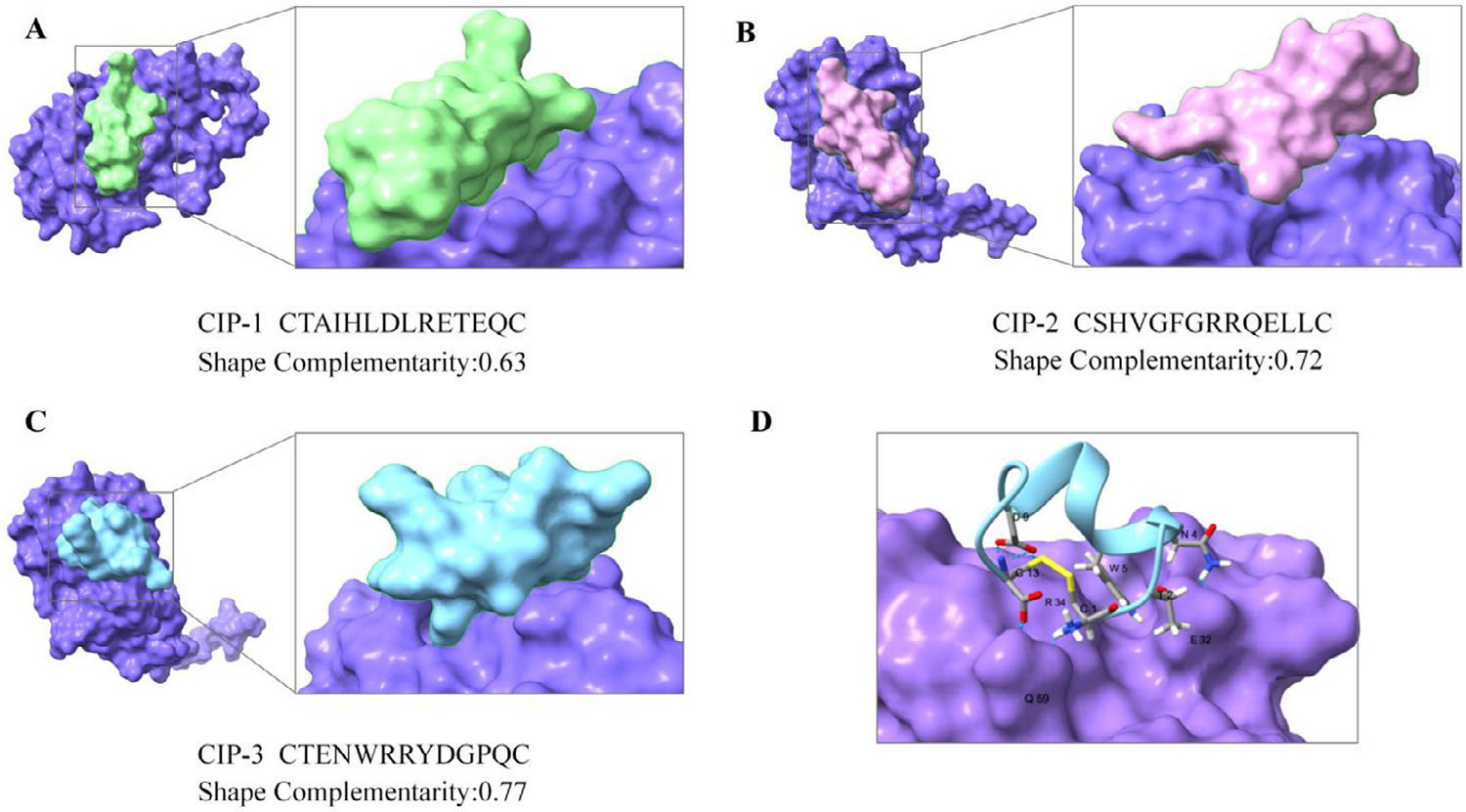
Interface complementarity and binding interactions of CIP peptides with CD28. (A–C) Surface representations illustrating the interface shape complementarity between CD28 and CIP‐1 (A), CIP‐2 (B), and CIP‐3 (C), with each complex corresponding to a distinct Sc value. (D) Binding interactions between CIP‐3 and CD28.

Experimental results confirmed that both CIP‐2 and CIP‐3 exhibited specific binding activity against CD28. Notably, the superior binding activity of CIP‐3 was mainly attributed to its outstanding geometric complementarity with the target binding interface. Unlike the flexible β‐sheet conformation adopted by CIP‐2, CIP‐3 (Figure 4A–D) forms a stable α‐helical structure, which confers a strong conformational pre‐organization effect and effectively reduces the entropy penalty upon target binding. This rigid cyclic backbone not only matches the spatial shape of the CD28 binding interface with high precision, but also drives the indole ring of Trp‐5 to deeply insert into the hydrophobic pocket of the target, optimizing hydrophobic packing interactions and significantly improving the overall binding affinity, as shown in the Figure 4D.

The absence of detectable binding for CIP‐1 provides an internal specificity control, arguing against nonspecific interactions driven solely by peptide cyclization or physicochemical properties. Instead, the observed affinity hierarchy supports a model in which effective CD28 engagement requires precise sequence‐encoded surface complementarity combined with conformational preorganization imposed by cyclization. Consistent with this, further quantitative analysis showed that the shape complementarity (Sc) value of CIP‐1(Figure 4A) was only 0.63, which was significantly lower than that of active peptides CIP‐2 (Figure 4B, Sc = 0.72) and CIP‐3 (Figure 4C, Sc = 0.77). Surface mode visualization revealed that although CIP‐1 formed local hydrogen bonds, obvious geometric gaps existed between CIP‐1 and the CD28 binding pocket, leading to poor interface packing. In protein–peptide interactions, such incomplete packing prevents full desolvation of interfacial water molecules, and the resulting entropy penalty offsets the energetic gain from hydrogen bonding. Collectively, these findings demonstrate that compact, disulfide‐constrained peptides can achieve nanomolar binding affinity to a costimulatory receptor characterized by a shallow extracellular interface.

### 2.3 CIP‐3 Disrupts CD28‐Ligand Interactions

**FIGURE 5:**
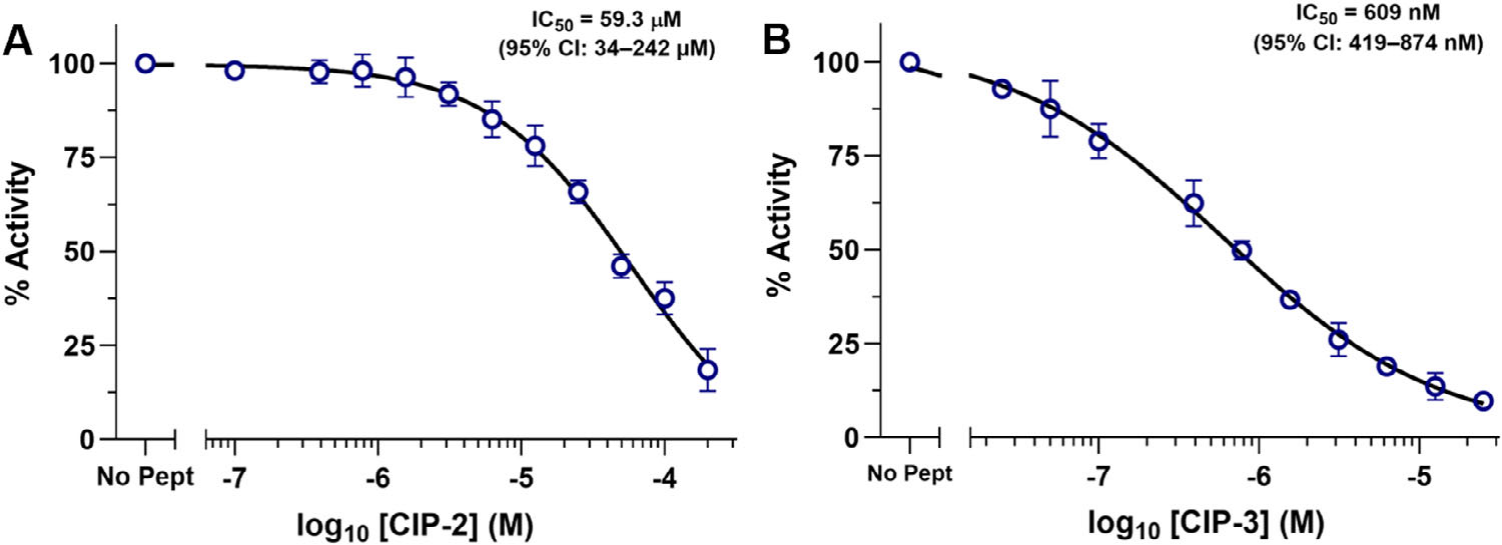
Cyclic peptides inhibit CD28‐CD80 interactions. Functional inhibition of CD28‐ligand engagement by cyclic peptides was assessed using a ligand‐binding inhibition assay in which peptide‐mediated blockade of biotinylated CD80 binding to immobilized recombinant human CD28 was quantified. (A) CIP‐2 inhibits CD28‐CD80 interaction in a concentration‐dependent manner with moderate potency. (B) CIP‐3 exhibits substantially enhanced inhibitory activity, yielding a well‐defined sigmoidal inhibition curve with submicromolar potency. Data represent mean ± SD from three independent experiments. IC_50_ values were determined by nonlinear regression analysis.

The ability of CD28‐binding cyclic peptides to functionally disrupt receptor‐ligand engagement was evaluated using a ligand‐binding inhibition assay. Consistent with biophysical measurements, both CIP‐2 and CIP‐3 inhibited CD28‐CD80 interactions in a concentration‐dependent manner, yielding well‐defined sigmoidal inhibition curves (Figure 5A,B). CIP‐2 displayed moderate inhibitory activity (IC_50_ = 59.3 µm, 95% CI: 34–242 µm), whereas CIP‐3 exhibited substantially greater potency (IC_50_ = 609 nm, 95% CI: 419–874 nm), representing an approximately 65‐fold improvement in functional activity.

The close agreement between MST‐derived binding affinities and functional inhibition supports a mechanism consistent with peptide engagement proximal to the CD28 ligand‐binding interface, resulting in inhibition of CD80 binding rather than nonspecific assay interference. Notably, the submicromolar potency of CIP‐3 demonstrates that a compact disulfide‐constrained peptide can effectively inhibit native costimulatory ligand engagement at an immune checkpoint receptor, a property traditionally achieved using biologic agents. Having established that cyclic peptides disrupt CD28‐CD80 interactions in a cell‐free system, we next evaluated whether this antagonism translates into modulation of CD28‐dependent signaling in cellular assays.

### 2.4 Cyclic Peptide Antagonists Suppress CD28‐Dependent Signaling in Cellular Systems

**FIGURE 6:**
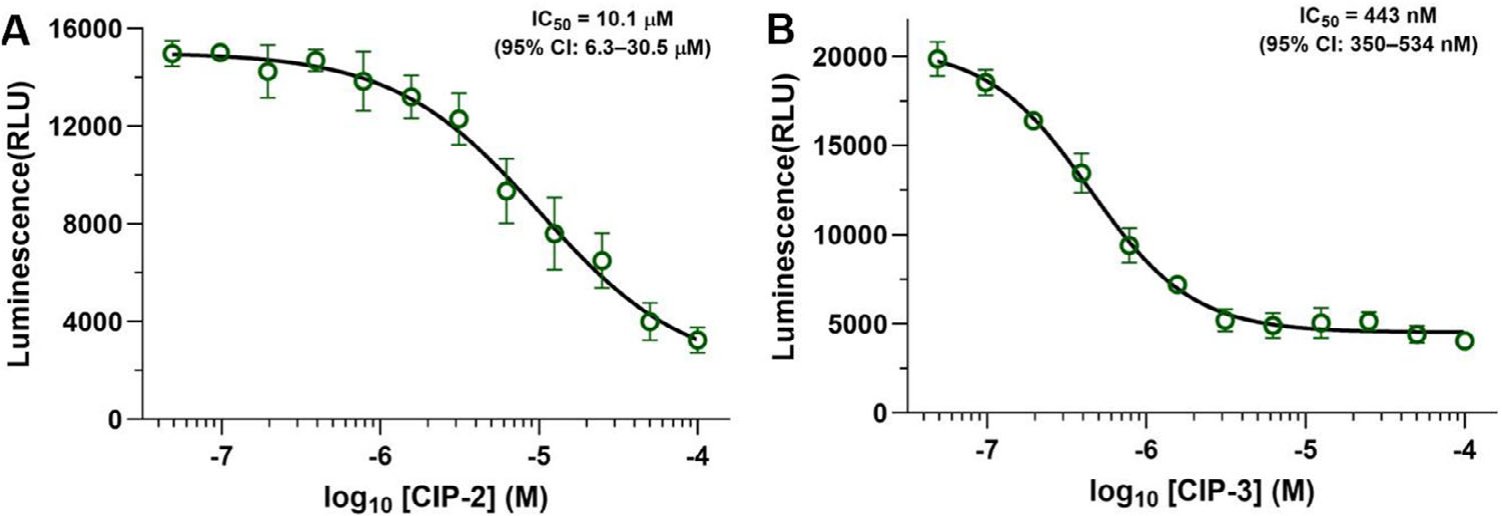
Cyclic peptides inhibit CD28‐dependent costimulatory signaling in cells. Functional antagonism of CD28 signaling was evaluated using a reporter assay in which Jurkat T cells were co‐cultured with antigen‐presenting cells to induce CD28‐dependent activation, quantified by an NFAT‐driven luciferase readout. (A) CIP‐2 inhibits CD28 signaling in a concentration‐dependent manner with moderate potency. (B) CIP‐3 exhibits substantially enhanced inhibitory activity, achieving submicromolar inhibition. Data represent mean ± SEM from at least three independent experiments. Concentration‐response curves were fitted using a four‐parameter logistic regression model.

The functional consequences of CD28 antagonism were evaluated using a luciferase reporter assay that quantitatively measures CD28 costimulatory signaling in Jurkat T cells co‐cultured with antigen‐presenting cells. Peptides capable of binding CD28 and disrupting CD28‐CD80 interactions suppressed reporter activity in a concentration‐dependent manner (Figure 6). CIP‐2 produced moderate inhibition (IC_50_ = 10.1 µm, 95% CI: 6.3–30.5 µm, Figure 6A), whereas CIP‐3 exhibited markedly enhanced potency with a submicromolar IC_50_ of 443 nm, 95% CI: 350–534 nm (Figure 6B). The similar submicromolar potency observed in the CD28‐CD80 ligand‐binding assay and the Jurkat CD28 reporter assay supports consistent functional inhibition across biochemical and cellular contexts. The modest difference in IC_50_ values likely reflects differences in assay format, including immobilized vs. cell‐surface receptor presentation, CD28 expression density on engineered Jurkat reporter cells, ligand density on aAPC/Raji cells, and downstream signal amplification.

The rank order of cellular activity closely mirrored trends observed in MST binding and ELISA‐based competition assays, supporting a direct relationship between peptide engagement of the CD28 extracellular domain and downstream signaling blockade. These findings demonstrate that disulfide‐constrained cyclic peptides can functionally antagonize CD28 signaling in a cellular environment, establishing CIP‐3 as a lead peptide with robust activity.

### 2.5 CIP‐3 Reproduces Biologic‐Like Immune Modulation in Primary Human Systems

**FIGURE 7:**
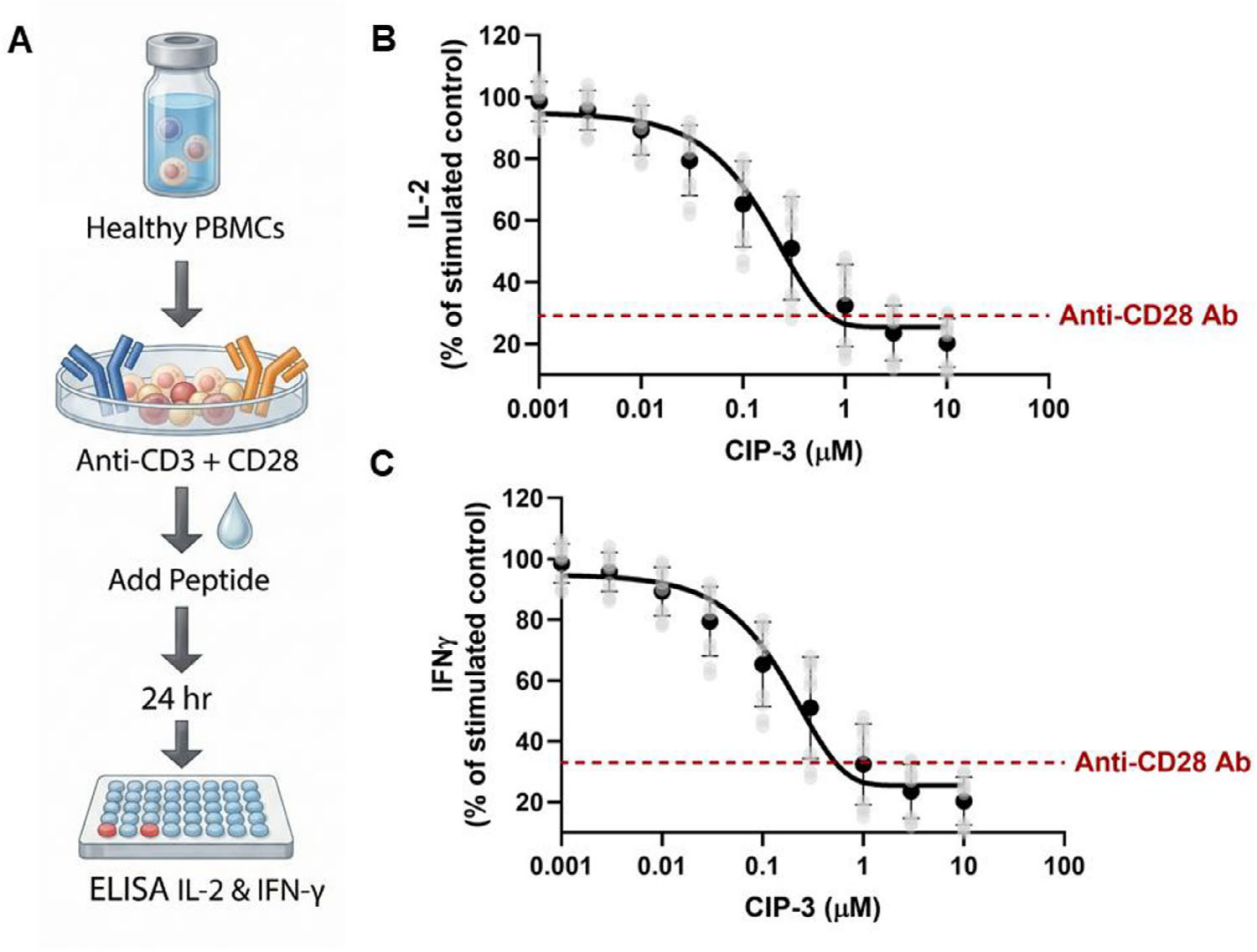
Cyclic CD28 peptide suppresses primary human T‐cell activation across independent donors. (A) Experimental schematic showing stimulation of peripheral blood mononuclear cells (PBMCs) from healthy donors with anti‐CD3/CD28 antibodies followed by treatment with cyclic peptide CIP‐3 and cytokine measurement after 24 h. (B, C) Concentration‐dependent inhibition of IL‐2 (B) and IFN‐γ (C) secretion by CIP‐3 across independent donors (*n* = 10). Light gray symbols represent individual donor responses, and black symbols indicate mean ± SEM. A benchmark anti‐CD28 blocking antibody (FR104, 10 µg mL^−1^) is shown for comparison (red dashed line).

To evaluate whether CIP‐3 could functionally modulate CD28‐mediated T‐cell activation in primary human systems, peripheral blood mononuclear cells (PBMCs) from independent healthy donors (*n* = 10 donors) were stimulated with anti‐CD3/CD28 antibodies in the presence of increasing concentrations of the cyclic peptide (Figure 7A). CIP‐3 produced a clear concentration‐dependent suppression of cytokine secretion, with progressive reductions observed in both IL‐2 and IFN‐γ levels (Figure 7B,C). Importantly, inhibitory activity was reproducible across a cohort of ten independent donors, despite expected inter‐individual variability in baseline cytokine responses, supporting a consistent pharmacologic effect across heterogeneous immune backgrounds.

At higher peptide concentrations, the magnitude of cytokine suppression approached that achieved with a benchmark anti‐CD28 blocking antibody (FR104) under matched experimental conditions, indicating that the cyclic peptide can produce biologically meaningful attenuation of CD28 co‐stimulatory signaling. Nonlinear regression analysis of the concentration‐response relationship revealed sub‐micromolar functional potency, further supporting effective engagement of the CD28 signaling axis.

### 2.6 CIP‐3 Exhibits no Intrinsic Agonist Activity and Mediates Reversible Checkpoint Modulation

**FIGURE 8:**
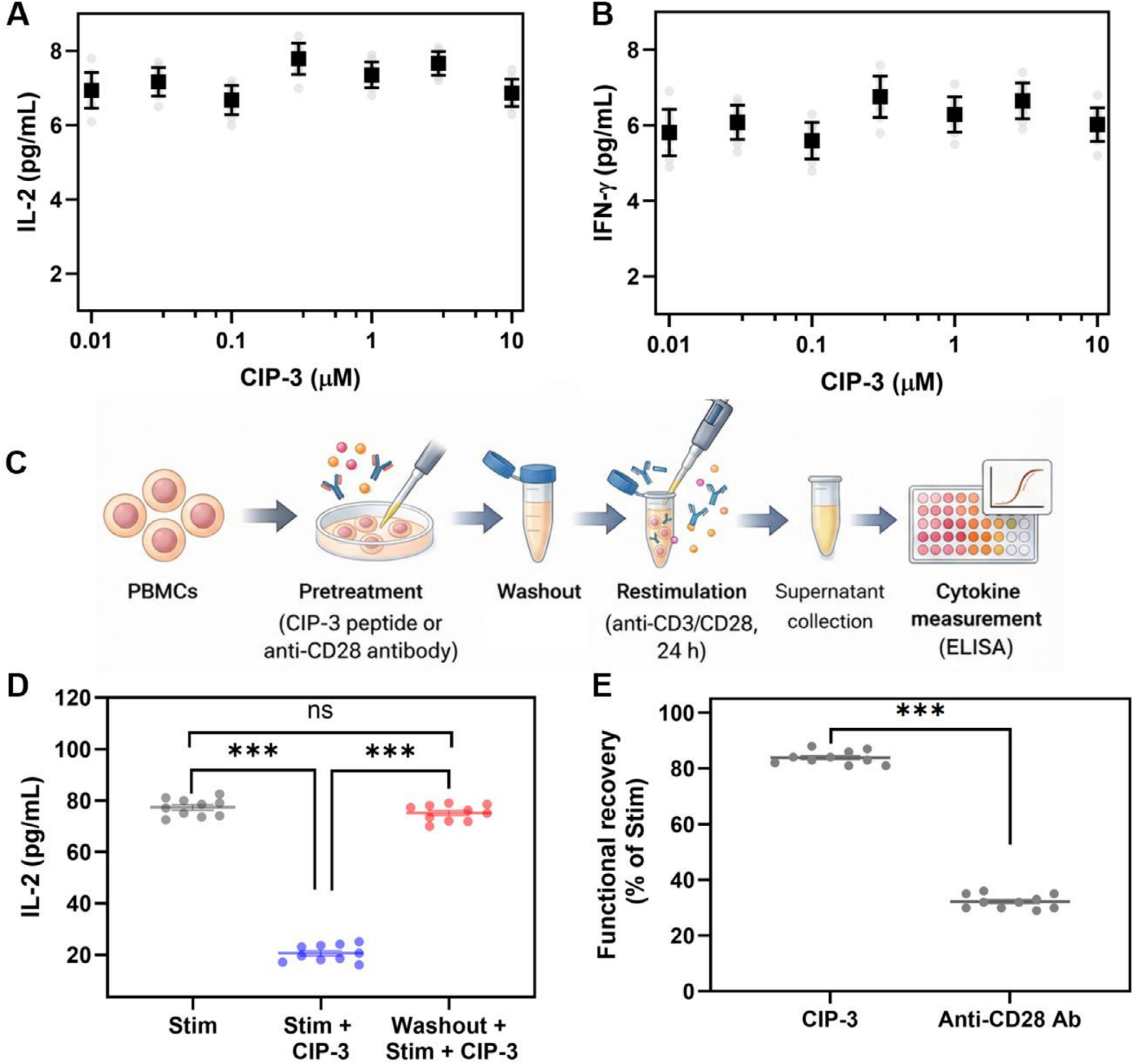
CIP‐3 lacks intrinsic agonist activity and mediates reversible modulation of CD28 signaling. (A, B) Primary PBMCs treated with CIP‐3 (0.01–10 µm) in the absence of stimulation show no increase in cytokine production relative to unstimulated controls, indicating the absence of intrinsic agonist activity. IL‐2 (A) and IFN‐γ (B) concentrations were measured by ELISA. (C) Experimental schematic illustrating pretreatment with CIP‐3 or anti‐CD28 antibody, washout (pre‐warmed RPMI medium), and subsequent restimulation with anti‐CD3/CD28 before cytokine measurement. (D) Functional recovery following inhibitor removal demonstrates rapid reversibility of peptide‐mediated inhibition (CIP‐3, 1 µm) compared with sustained inhibition following anti‐CD28 antibody treatment (10 µg/mL). (E) Quantification of functional recovery expressed as a percentage of stimulated control highlights substantially greater recovery following CIP‐3 washout relative to antibody treatment. Data represent mean ± SEM from independent donors (*n* = 10). Statistical analysis was performed using one‐way ANOVA with multiple comparisons unless otherwise indicated. ns, not significant; ^***^ *p* < 0.001.

Given historical safety concerns associated with CD28 targeting, we first evaluated whether CIP‐3 alone could induce immune activation in the absence of co‐stimulatory signaling. Treatment of primary PBMCs with CIP‐3 across the tested concentration range (0.01–10 µm) did not increase cytokine production relative to unstimulated baseline controls (Figure 8A,B), indicating the absence of intrinsic agonist activity. These findings suggest that receptor engagement by the cyclic peptide does not trigger unintended CD28 signaling and support a favorable functional safety profile compared with agonistic CD28 antibodies.

We next examined whether peptide‐mediated inhibition could be pharmacologically controlled through reversible receptor engagement. In washout experiments, PBMCs were preincubated with CIP‐3 (1 µm) or a benchmark anti‐CD28 antibody (FR104, 1 µg mL^−1^), followed by removal of unbound inhibitor before stimulation (Figure 8C). CIP‐3 and FR104 were used at concentrations that produced robust inhibition in the corresponding stimulation assay rather than as a molar‐equivalent comparison. This design allowed assessment of functional recovery after removal of unbound inhibitor under conditions of initial pathway suppression. Peptide‐treated cells exhibited rapid recovery of signaling activity, reaching approximately 85% of stimulated control levels following washout, whereas biologic‐mediated inhibition persisted with only approximately 30% recovery over the same period (Figure 8D,E). These results demonstrate that CIP‐3 engages CD28 in a transient and reversible manner, consistent with noncovalent ligand‐receptor interactions, in contrast to the prolonged receptor occupancy associated with antibody binding.

The rapid reversibility observed with CIP‐3 indicates that synthetic cyclic ligands can enable tunable modulation of CD28 signaling with temporal control distinct from antibody‐based approaches. Importantly, the combination of absent intrinsic agonism and controllable pharmacological reversibility suggests a therapeutic modality with a potentially improved safety window compared with conventional biologic checkpoint modulators. Results were reproducible across independent donors (*n* = 10), supporting robustness across heterogeneous immune backgrounds.

### 2.7 CIP‐3 Suppresses CD28‐Mediated Cytokine Production in T Cells Derived From Patients With Ulcerative Colitis

**FIGURE 9:**
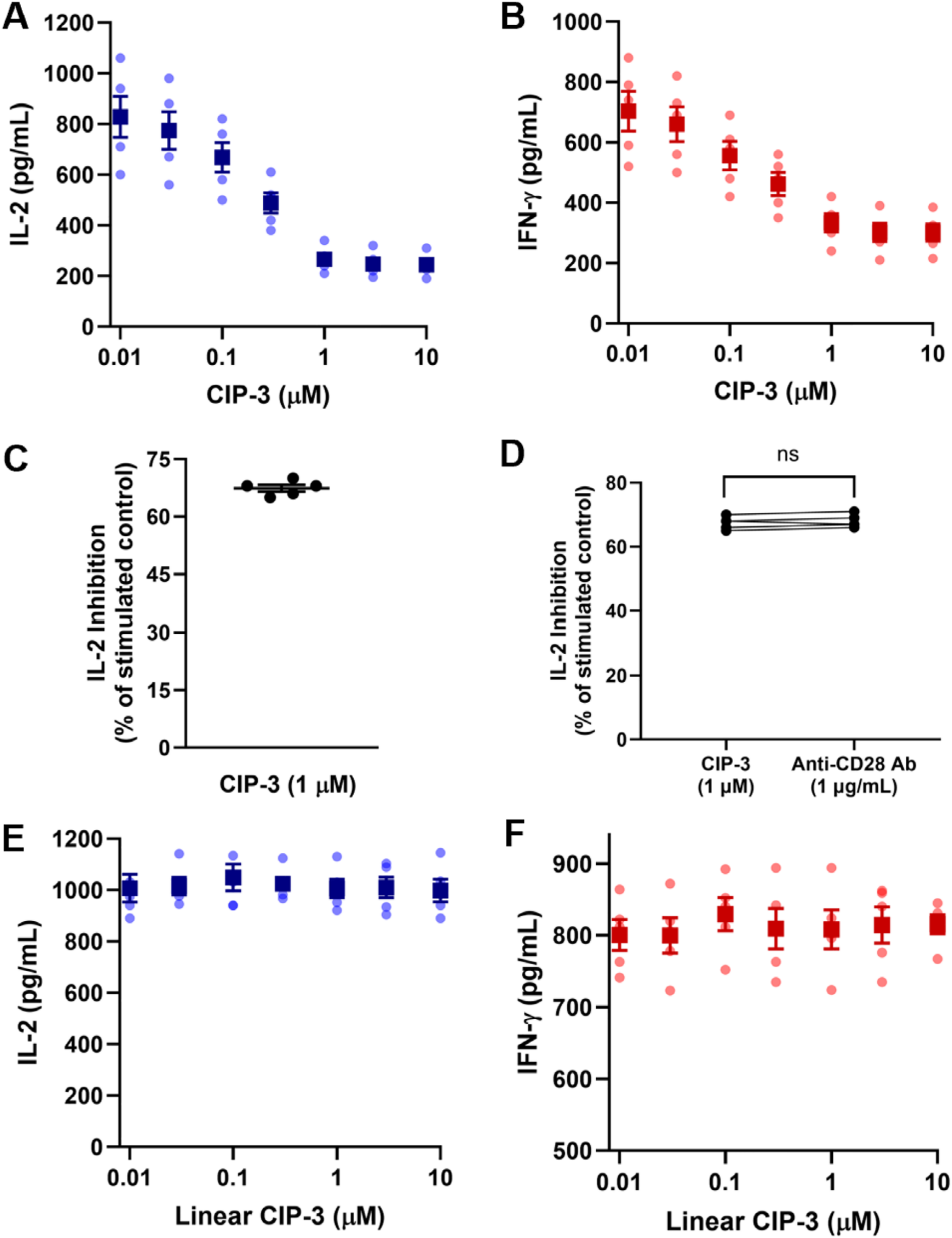
CIP‐3 suppresses CD28‐mediated cytokine production in PBMCs from patients with ulcerative colitis with efficacy comparable to a benchmark biologic. (A, B) PBMCs obtained from donors with ulcerative colitis (*n* = 5) were stimulated with anti‐CD3/CD28 and treated with increasing concentrations of CIP‐3 (0.01–10 µm). CIP‐3 produced dose‐dependent inhibition of IL‐2 (A) and IFN‐γ (B) secretion measured by ELISA. Data points represent individual donors with mean ± SEM. (C) IL‐2 inhibition across independent patient samples at 1 µm CIP‐3 demonstrates consistent activity in disease‐derived primary immune cells. (D) Comparison of peptide‐mediated and antibody‐mediated inhibition across matched patient samples shows that CIP‐3 (1 µm) achieves levels of IL‐2 inhibition comparable to those obtained with an anti‐CD28 monoclonal antibody (10 µg/mL) (paired two‐tailed *t*‐test, ns). (E, F) A non‐cyclized linear analog of CIP‐3 (“Linear CIP‐3”) was evaluated under identical experimental conditions to assess the contribution of conformational constraint to CD28 modulation. In contrast to cyclic CIP‐3, the linear peptide did not suppress IL‐2 (E) or IFN‐γ (F) production across the tested concentration range, supporting the importance of cyclization for functional activity.

To determine whether cyclic peptides can achieve functionally meaningful modulation of T‐cell co‐stimulation in disease‐relevant human systems, we evaluated CIP‐3 using PBMCs obtained from patients with ulcerative colitis (*n* = 5). Upon anti‐CD3/CD28 stimulation, CIP‐3 induced a concentration‐dependent attenuation of cytokine production, suppressing both IL‐2 and IFN‐γ secretion across donors (Figure 9A,B). Notably, inhibition plateaued at micromolar concentrations, consistent with saturable but incomplete pathway modulation rather than binary receptor blockade.

This graded suppression profile contrasts with the fixed amplitude typically associated with monoclonal antibody‐mediated inhibition and suggests that peptide engagement permits pharmacologic tuning of co‐stimulatory signaling intensity. Across independent patient samples, CIP‐3 reproducibly attenuated IL‐2 production at a pharmacologically relevant concentration (1 µm), with limited inter‐donor variability (Figure 9C), indicating robust activity within heterogeneous inflammatory immune environments.

To benchmark functional efficacy, we directly compared peptide‐mediated modulation with a clinically relevant anti‐CD28 monoclonal antibody (FR104) in matched patient samples. CIP‐3 produced a similar trend in IL‐2 suppression relative to antibody treatment under the tested conditions, with no statistically significant difference between conditions (Figure 9D). Collectively, these findings support the feasibility that CD28 co‐stimulatory signaling can be modulated in a tunable, pharmacologically controllable manner using a non‐antibody ligand, supporting the feasibility of peptide‐based immune checkpoint modulation in inflammatory disease.

To further assess the contribution of conformational constraint to CD28 modulation, we synthesized and evaluated a non‐cyclized linear analog of CIP‐3 (“Linear CIP‐3”) under identical experimental conditions in primary PBMC assays. In contrast to cyclic CIP‐3, the linear peptide failed to suppress IL‐2 or IFN‐γ production across the tested concentration range (Figure 9E,F), despite sharing the same primary amino acid sequence. These findings support the importance of cyclization and conformational preorganization for efficient functional engagement of the CD28 signaling axis and further argue against nonspecific peptide‐mediated immunosuppression.

### 2.8 CIP‐3 Demonstrates Favorable Acute In Vivo Tolerability Without Detectable Systemic Cytokine Release

Given the historical safety concerns associated with CD28‐targeting agents, we next performed an initial in vivo tolerability assessment of CIP‐3 in healthy mice (Figure S9A–F). Animals received subcutaneous administration of CIP‐3 at the therapeutically active dose used in the colitis studies (5 mg/kg) or at a higher dose (10 mg/kg), and were monitored for clinical signs of toxicity, body weight changes, and systemic cytokine release.

CIP‐3 was generally well tolerated under the tested conditions, with no overt behavioral abnormalities or treatment‐associated body weight loss observed during the monitoring period (Figure S9B). To assess the potential for acute immune activation, serum cytokines were quantified following peptide administration. In contrast to the cytokine release historically associated with agonistic CD28 antibodies, CIP‐3 did not induce detectable elevations in circulating IL‐6, TNF‐α, IFN‐γ, or IL‐2 levels relative to vehicle‐treated controls (Figure S9C–E).

To further evaluate systemic tolerability, hematological and serum chemistry parameters were assessed at study termination. No significant alterations were observed in major hematologic indices or markers of hepatic and renal function following CIP‐3 administration (Figure S9F). Collectively, these findings support the absence of overt acute systemic toxicity or cytokine‐release activity under the tested conditions and further distinguish peptide‐mediated CD28 modulation from agonistic CD28‐directed biologic approaches.

### 2.9 CIP‐3 Demonstrates Dose‐Dependent Therapeutic Efficacy in a T‐Cell Transfer Model of Colitis

**FIGURE 10:**
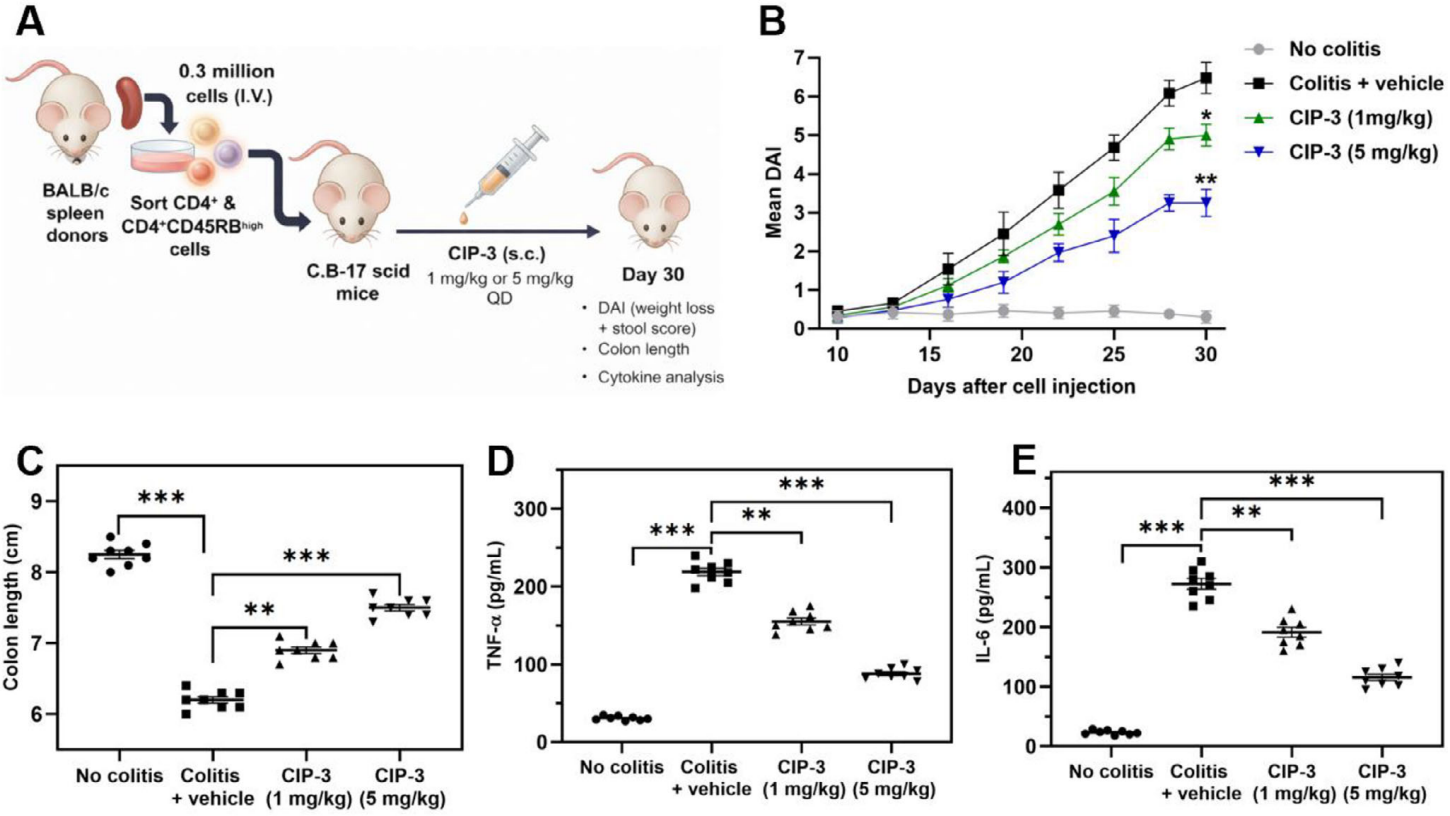
CIP‐3 confers dose‐dependent therapeutic efficacy in a T‐cell transfer model of colitis. (A) Schematic representation of the adoptive CD4^+^CD45RB^high^ T‐cell transfer model of chronic colitis. CD4^+^CD45RB^high^ T cells were isolated from BALB/c donor spleens and intravenously transferred into C.B‐17 scid recipient mice. Beginning after cell transfer, mice received daily subcutaneous administration of CIP‐3 (1 or 5 mg/kg) or vehicle. Disease progression was monitored for 30 days. No‐colitis controls received daily vehicle administration without T‐cell transfer. Endpoints included disease activity index (DAI), colon length, and serum cytokine analysis. (B) CIP‐3 reduced disease severity in a dose‐dependent manner. DAI (combined weight loss and stool score) was assessed longitudinally. Vehicle‐treated mice developed progressive colitis, whereas CIP‐3 treatment significantly attenuated disease progression, with greater efficacy observed at 5 mg/kg. (C) CIP‐3 mitigated colonic shortening associated with inflammation. Colon length was measured on day 30. Vehicle‐treated mice exhibited marked shortening relative to no‐colitis controls, whereas CIP‐3 treatment significantly restored colon length in a dose‐dependent manner. (D‐E) CIP‐3 suppressed systemic inflammatory cytokines. Serum TNF‐α (D) and IL‐6 (E) levels were quantified by ELISA at study termination. Vehicle‐treated mice showed elevated cytokine concentrations, while CIP‐3 significantly reduced TNF‐α and IL‐6 levels, with stronger suppression at 5 mg/kg. Data represent mean ± SEM (*n* = 8 per group). Statistical analysis was performed using one‐way ANOVA with Tukey's multiple comparison test for endpoint analyses and two‐way ANOVA for longitudinal DAI measurements. ^*^ *p* < 0.05, ^**^ *p* < 0.01, ^***^ *p <* 0.001 vs. colitis + vehicle group.

To evaluate the in vivo therapeutic potential of CIP‐3, we employed the adoptive CD4^+^CD45RB^high^ T‐cell transfer model of chronic colitis in C.B‐17 scid mice (Figure 10A). This model recapitulates key features of human IBD, including progressive weight loss, diarrhea, colonic shortening, and elevated systemic inflammatory cytokines (42‐44). We first established functional cross‐reactivity with murine CD28 to ensure translational relevance of the in vivo model. In primary murine splenocytes stimulated with anti‐CD3/CD28, CIP‐3 dose‐dependently suppressed T‐cell activation and cytokine production, confirming engagement of murine CD28 signaling (Figure S6).

Before in vivo efficacy studies, we characterized the pharmacokinetic (PK) and metabolic stability profile of CIP‐3. In mouse plasma, CIP‐3 demonstrated high stability, with >85% parent compound remaining after 6 h at 37°C. In liver microsomes, CIP‐3 exhibited low intrinsic clearance in both mouse (Cl_int_ = 6.8 µL/min/mg protein) and human (Cl_int_ = 4.9 µL/min/mg protein) systems, corresponding to microsomal half‐lives of 102 min (mouse) and 138 min (human), respectively. Following a single subcutaneous administration in C57BL/6 mice, CIP‐3 displayed: C_max_ (5 mg/kg): 3.4 ± 0.6 µg/mL, C_max_ (1 mg/kg): 0.72 ± 0.11 µg/mL, T_max_: 0.5–1 h, Terminal half‐life (t_1/2_): 3.9 ± 0.4 h, apparent clearance (CL/F): 0.42 L/h/kg, and AUC_0‐24 h_ (5 mg/kg): 12.8 ± 1.9 µg·h/mL. Importantly, plasma concentrations remained above the in vitro functional concentrations for CD28 inhibition (≈ 500 nm) for approximately 10–12 h at 5 mg/kg and 4–6 h at 1 mg/kg. Plasma concentration‐time curves following subcutaneous administration of CIP‐3 are provided in Figure S8. These exposure levels and half‐life parameters supported a once‐daily subcutaneous dosing regimen and justified evaluation of 1and 5 mg/kg in the T‐cell transfer colitis model.

Vehicle‐treated mice developed progressive colitis characterized by increasing disease activity index (DAI) scores over 30 days (Figure 10B). In contrast, CIP‐3 significantly reduced disease severity in a dose‐dependent manner. Mice treated with 1 mg/kg CIP‐3 exhibited partial attenuation of DAI progression, whereas 5 mg/kg treatment produced a more pronounced reduction in disease burden, demonstrating clear pharmacodynamic dose responsiveness. Chronic inflammation in vehicle‐treated mice resulted in marked colon shortening at study termination (Figure 10C). Treatment with CIP‐3 significantly restored colon length relative to vehicle controls. The 1 mg/kg group showed partial structural rescue, while the 5 mg/kg treatment more robustly preserved colon length, consistent with the observed improvements in clinical scores.

To assess the impact of CIP‐3 on inflammatory mediators, serum cytokine levels were quantified at day 30. Vehicle‐treated colitic mice displayed elevated TNF‐α and IL‐6 concentrations (Figure 10D, E). CIP‐3 significantly reduced both cytokines in a dose‐dependent manner, with greater suppression observed at 5 mg/kg. These findings indicate that CIP‐3 dampens pathogenic T‐cell‐driven inflammatory responses in vivo. Together, these data demonstrate that CIP‐3 confers dose‐dependent therapeutic benefit in a T‐cell‐mediated model of colitis, with concordant improvements in clinical disease indices, anatomical preservation of colon length, and systemic cytokine suppression. The in vivo efficacy is supported by validated murine CD28 engagement and PK properties that sustain target‐relevant exposure under once‐daily subcutaneous administration.

## 3 Discussion

The present study establishes cyclic peptides as a controllable synthetic modality for targeting immune costimulatory receptors traditionally addressed using biologic agents. Through an AI‐guided discovery strategy, we identified a disulfide‐constrained cyclic peptide capable of nanomolar binding to the extracellular domain of CD28 and demonstrated that this molecule achieves functional suppression of T‐cell activation across primary human immune systems. Importantly, this synthetic ligand exhibits reversible pharmacology, absence of intrinsic agonist activity, and dose‐dependent therapeutic efficacy in vivo. These findings provide proof of principle that compact cyclic scaffolds can function as systemically active modulators of immune checkpoint signaling.

Targeting costimulatory receptors with synthetic ligands has historically been constrained by structural considerations. The extracellular domains of receptors such as CD28 present shallow protein‐protein interaction surfaces that lack the deep binding pockets typically exploited by small molecules. Our results demonstrate that conformationally constrained cyclic peptides can effectively engage these interfaces with nanomolar affinity and disrupt receptor‐ligand interactions. By presenting preorganized interaction motifs across extended surfaces, cyclic peptides can achieve high surface complementarity while retaining the synthetic tractability and modular optimization potential of chemically defined ligands.

A defining feature of this approach is pharmacologic controllability. In contrast to antibody‐based checkpoint blockade, which is often characterized by prolonged receptor occupancy and sustained signaling modulation, peptide‐mediated antagonism of CD28 was rapidly reversible following washout. The absence of intrinsic agonist activity further distinguishes this synthetic ligand from antibody‐based strategies that carry the risk of unintended receptor activation. Together, these properties suggest that cyclic peptide antagonists may enable exposure‐dependent tuning of immune signaling intensity, providing temporal control over immune modulation. An additional consideration is the potential for cross‐reactivity with related immune checkpoint receptors that share the CD80/CD86 ligand system, particularly CTLA‐4. Because CTLA‐4 mediates inhibitory immune signaling, unintended receptor engagement could theoretically produce distinct immunological consequences. Although the current computational and functional data support modulation of CD28‐dependent signaling, direct receptor selectivity profiling against CTLA‐4 and related checkpoint receptors will be important in future mechanistic studies.

The translational relevance of this modality is supported by activity across multiple biological systems. CIP‐3 reproducibly suppressed cytokine production in PBMCs derived from independent healthy donors and from patients with ulcerative colitis, demonstrating functional robustness across heterogeneous immune backgrounds. PK characterization revealed favorable plasma stability and sustained systemic exposure following subcutaneous administration, enabling target‐relevant engagement in vivo. In a T‐cell transfer model of chronic colitis, cyclic peptide treatment produced dose‐dependent reductions in disease activity, preservation of colon length, and suppression of systemic inflammatory cytokines, establishing proof of concept that synthetic checkpoint modulation can translate into therapeutic benefit.

Several considerations warrant further investigation. Although the proposed interaction interface is supported by computational modeling together with orthogonal biophysical and functional assays, future structural studies, including mutagenesis and high‐resolution co‐structural approaches, will further define the molecular details of CIP‐3 engagement with CD28. Additionally, the primary PBMC activation studies employed anti‐CD28 antibody‐mediated co‐stimulation rather than native CD80/CD86 ligand engagement. Although the combined biochemical, computational, and cellular data support peptide interaction proximal to the ligand‐binding interface, future studies using native ligand‐dependent systems together with mutagenesis and structural approaches will further clarify the precise mechanism and epitope of CD28 modulation by CIP‐3. Moreover, the primary PBMC activation studies employed anti‐CD28 antibody‐mediated co‐stimulation rather than native APC‐T cell interactions. Although this approach enabled controlled evaluation of downstream CD28‐dependent signaling, future studies using APC‐mediated CD80/CD86 co‐stimulation systems will further clarify the ability of CIP‐3 to modulate the physiological CD28‐B7 signaling axis in cellular contexts. An additional limitation is that PK characterization was performed in immunocompetent C57BL/6 mice, whereas therapeutic efficacy studies were conducted in immunodeficient C.B‐17 SCID mice. Consequently, the PK‐PD relationship within the efficacy model is inferred rather than directly measured and will require future characterization directly within the disease model.

Although the present in vivo studies demonstrate dose‐dependent therapeutic responsiveness across multiple disease‐associated endpoints, additional studies incorporating histopathological analysis, immune cell profiling, and direct comparisons with established therapeutic agents will further strengthen the translational assessment of cyclic peptide‐mediated CD28 modulation. Disease activity scoring included semi‐subjective parameters and was not performed in a blinded manner. Although objective endpoints, including colon length and systemic cytokine measurements, independently supported therapeutic activity, future studies incorporating blinded assessment, histopathological evaluation, immune cell profiling, and benchmarking against established therapeutic agents will further strengthen translational interpretation. In addition, the present ligand‐binding studies focused on CD80‐mediated CD28 engagement, whereas CD86 can represent a dominant physiological ligand in certain immune contexts. Future studies evaluating the effects of CIP‐3 on CD28‐CD86 interactions will further refine the understanding of ligand‐selective checkpoint modulation.

In summary, these findings establish AI‐designed cyclic peptides as a viable synthetic modality for immune checkpoint modulation. By combining structural complementarity to protein–protein interaction interfaces with exposure‐dependent pharmacologic control, cyclic peptides provide a bridge between small molecules and biologics and offer a foundation for next‐generation immunotherapies targeting costimulatory receptors.

## 4 Materials and Methods

### 4.1 HighPlay Framework

The HighPlay framework employs reinforcement learning as its core strategy and mainly integrates three key modules: the Monte Carlo tree search (MCTS) reinforcement learning algorithm, a Transformer‐based policy‐value network, and the HighFold model. MCTS shows unique advantages in biological sequence design, as it can effectively balance exploration and exploitation within a large, high‐dimensional discrete search space, thereby converging toward better global solutions. In the cyclic peptide sequence optimization pipeline, MCTS performs iterative sequence search, the Transformer policy‐value network provides prior guidance for MCTS decision‐making, and HighFold acts as a high‐precision proxy model to generate reward signals and support iterative retraining of the policy‐value network.

For a cyclic peptide containing L amino acid residues, HighPlay defines its state space as an L × 20 binary matrix, where rows represent residue positions and columns represent the 20 types of natural amino acids. The action space is a one‐dimensional vector obtained by flattening this matrix. During the simulation rollout phase, the model introduces sequence perturbation by sampling a single position from the action space to perform a single‐point mutation on the cyclic peptide. State updates continue iteratively until a predefined termination condition is triggered. The first condition is a reward threshold constraint: if the current state reward is lower than the previous state reward, the mutation is considered to provide no performance gain. The second condition is a sequence repetition constraint: if the mutated peptide sequence matches any previously recorded state, the action is deemed invalid, and the current iteration terminates.

This framework was implemented on a computing platform running Ubuntu 20.04.6. The maximum number of MCTS iterations was set to 2500, and convergence was defined as the policy neural network reaching a maximum of 800 training epochs. The hardware configuration included dual AMD EPYC 7K62 48‐Core processors, four NVIDIA RTX 4090 GPUs (96 GB total GDDR6X memory), 503 GB of RAM, and 6 TB of storage.

### 4.2 AI‐Guided Cyclic Peptide Design

A series of data preprocessing steps was performed before model‐based design to ensure the compatibility of Protein Data Bank (PDB) files with our computational workflow. The design of CD28 binders was based on the PDB structure 1YJD; the amino acid sequence of chain C was extracted using a custom Python script and subsequently used as the input sequence of the CD28 target for the Highplay platform [38].

The binding epitope of CD28 consists of a relatively flat β‐sheet domain. Given our focus on the interfacial binding characteristics of peptide‐target complexes, a threshold of interface predicted local distance difference test (i_pLDDT) > 0.85 [40, 41] was applied in this study to ensure the structural accuracy of the peptide‐CD28 binding interface. On the premise of validating the structural rationality of the binding interface, further screening was conducted with the criteria of number of hydrogen bonds (Hbonds) > 8 in the whole complex and interface hydrogen bonds (i_Hbonds) > 3 at the peptide‐target contact site. These criteria were set to guarantee sufficient side chain‐mediated intermolecular interactions between cyclic peptides and the CD28 surface, thus identifying candidate molecules with high binding affinity. Based on the aforementioned screening metrics, we established a standardized computational pipeline for the design and primary screening of CD28‐targeting cyclic peptides. For the pre‐screened cyclic peptide candidates, we performed in‐depth structural analyses, including the characterization of side chain interaction modes with the CD28 target, the assessment of binding site accessibility, and a comprehensive evaluation of chemical synthetic feasibility. Ultimately, through a rigorous multi‐stage filtering process, three final candidates were prioritized from an initial library of 420 designed sequences for in vitro experimental validation, namely CIP‐1 (CTAIHLDLRETEQC), CIP‐2 (CSHVGFGRRQELLC), and CIP‐3 (CTENWRRYDGPQC). All three candidates exhibited favorable computational metrics, displayed a high degree of structural complementarity to the CD28 binding interface, and thus possessed considerable potential for high CD28 binding affinity.

### 4.3 Peptide Synthesis and Characterization

Peptide synthesis was carried out by solid phase peptide synthesis (SPPS) via the Fmoc/tBu strategy using an automated microwave peptide synthesizer—Liberty BLUE (CEM, Matthews, NC, USA). 424 mg Rink Amide ProTide Resin (0.59 mmol/g; CEM, Matthews, NC, USA) was used to synthesize each peptide. During the synthesis, each of the amino acids was coupled twice, using a fivefold excess of the amount of resin deposition. The following solutions were used during the synthesis: OxymaPure/DIC as coupling reagents, 20% piperidine in DMF for Fmoc‐deprotection, and DMF to wash between the deprotection and coupling steps. Synthesized peptides were cleaved from the resin using a mixture containing: 88% TFA, 5% H2O, 5% phenol, and 2% TIPSI (10 mL of solution was used for 424 mg of resin). Crude peptides were precipitated with cold diethyl ether, decanted, and lyophilized. For purification, the peptides were dissolved in water with the addition of a 10‐fold molar excess of dithiothreitol (DTT) over free sulfhydryl groups and incubated for 30 min at 60°C. Peptides were purified by reversed phase HPLC, XBridge Prep C18 column (19 × 150 mm, 5 µm, Waters, MA, USA), flow rate 20 mL/min, 10 min run, 5%–60% acetonitrile in water with 0.1% formic acid. The purity and mass spectra of the final products were analyzed using an ACQUITY HPLC system equipped with an SQ Detector 2 and an XBridge Prep C18 analytical column (4.6 × 150 mm, 5 µm, Waters, MA, USA), employing a linear gradient from 5% to 100% acetonitrile in water containing 0.1% formic acid over 10 min. Oxidation of the peptides was performed using compressed air. The peptide was dissolved in H2O and methanol (1:9, v:v), at a concentration of about 40 mg/L, and the pH was adjusted and kept between 8 and 9 using ammonia. The solution was stirred at room temperature for 7 days, and compressed air was run through the solution. After this time, the solvents were evaporated, and the peptides were lyophilized. Reaction progress was checked using an analytical ACQUITY HPLC system equipped with an SQ Detector 2. After this process, the peptides were purified again using the same ACQUITY HPLC system on a XBridge Prep C18 column (19 × 150 mm, 5 µm, Waters, MA, USA). A linear gradient 5%–60% acetonitrile in water with 0.1% formic acid over 10 min was used.

### 4.4 Recombinant Proteins

Recombinant human CD28 extracellular domain (ECD) was obtained from Acro Biosystems (Catalog# CD8‐H52H3) and reconstituted according to manufacturer's instructions. Proteins were stored in aliquots at −80°C to avoid repeated freeze‐thaw cycles. Recombinant CD28 ECD quality and expected molecular weight were verified based on manufacturer‐provided quality‐control documentation. Functional integrity of the CD28 preparation was further supported by its robust binding to biotinylated CD80 in the CD28‐CD80 competition assay.

### 4.5 Microscale Thermophoresis (MST) Binding Assays

The binding affinities of cyclic peptides to the human CD28 ECD were determined by MST in spectral shift mode. His‐tagged recombinant human CD28 ECD (Acro Biosystems) was fluorescently labeled using the RED‐tris‐NTA second‐generation dye (NanoTemper Technologies) according to the manufacturer's protocol and diluted to a final concentration of 100 nm in assay buffer consisting of PBS (pH 7.0) supplemented with 0.005% Tween‐20.

Cyclic peptides were prepared as serial dilutions in assay buffer and mixed 1:1 with labeled CD28 protein. Samples were incubated for 15 min at room temperature in the dark before measurement. MST experiments were performed at 25°C using a Monolith X instrument (NanoTemper Technologies) and loaded into Monolith Capillaries.

Fluorescence was recorded at 670 and 650 nm, and normalized fluorescence (Fnorm) was calculated as the ratio F670/F650. Dissociation constants (Kd) were obtained by fitting concentration‐response curves by nonlinear regression fitting using a 1:1 binding model (GraphPad Prism 10). Reported values represent mean ± SD from at least three independent experiments.

### 4.6 Isothermal Titration Calorimetry (ITC)

Thermodynamic characterization of peptide binding to the CD28 extracellular domain was performed using isothermal titration calorimetry (ITC). Experiments were conducted at 25°C using a NanoITC calorimeter (TA Instruments). Recombinant human CD28 protein was prepared in PBS buffer (pH 7.4) and loaded into the calorimetric cell at a final concentration of 6 µm.

Peptide solutions were prepared in the same buffer and loaded into the injection syringe at an initial concentration of 85 µm. A series of 32 sequential injections (1.49 µL per injection) were titrated into the CD28 solution under constant stirring at 250 rpm, with sufficient spacing between injections to allow the signal to return to baseline. Samples were degassed before measurements.

Heat changes upon each injection were recorded as a function of time to generate raw thermograms. The integrated heat for each injection was plotted against the molar ratio of peptide to CD28 to obtain binding isotherms. Thermodynamic parameters, including the Kd, enthalpy change (ΔH), entropy change (ΔS), and binding stoichiometry (n), were determined by fitting the data to a one‐site binding model using NanoAnalyze software (TA Instruments).

### 4.7 CD28‐Ligand Competition Assays

Inhibition of CD28‐CD80 interaction was evaluated using a ligand‐binding inhibition ELISA assay with the CD28:B7‐1 [Biotinylated] Inhibitor Screening Kit (BPS Bioscience, Cat. #72007), following the manufacturer's protocol with minor modifications. Briefly, 96‐well plates were coated with recombinant human CD28 (2 µg/mL in PBS, 50 µL per well) and incubated overnight at 4°C. Plates were washed with Immuno Buffer and blocked with Blocking Buffer for 1 h at room temperature.

Serial dilutions of cyclic peptides were first added to CD28‐coated wells and incubated for 1 h at room temperature to allow peptide binding. Biotinylated CD80 (5 ng/µL) was then added to the wells and incubated for an additional 1 h to assess inhibition of ligand binding to peptide‐occupied CD28. Wells lacking CD28 coating served as ligand controls, while wells treated with inhibitor buffer alone were used as negative controls.

After washing, plates were incubated with streptavidin‐HRP (1:1000 dilution in Blocking Buffer) for 1 h at room temperature. Chemiluminescent substrate was added, and luminescence was immediately measured using an Infinite M1000 Pro microplate reader (Tecan, Switzerland). IC_50_ values were determined by fitting concentration‐response curves using a four‐parameter logistic regression model in GraphPad Prism 10. All experiments were performed in triplicate.

### 4.8 Cell‐Based CD28 Signaling Reporter Assays

Functional inhibition of CD28‐mediated costimulatory signaling was assessed using the CD28 Blockade Bioassay (Promega, Cat. #JA6101). Jurkat CD28 Effector Cells (2 × 10^4^ cells per well) were seeded in white 96‐well plates and pre‐incubated with serial dilutions of cyclic peptides (10‐point, 1:1 dilution series starting at 200 µm, final 1% DMSO) for 1 h at room temperature. An anti‐CD28 control antibody (Promega, Cat. #K1231) was included as a positive control.

Following peptide pre‐incubation, aAPC/Raji cells (2 × 10^4^ cells per well) were added, and co‐cultures were incubated for 5 h at 37°C in a humidified atmosphere containing 5% CO_2_. After incubation, Bio‐Glo Luciferase Reagent (Promega) was added according to the manufacturer's instructions, and luminescence was measured using a GloMax Discover System (Promega).

Dose‐response curves were generated using GraphPad Prism 10 by fitting data to a four‐parameter logistic regression model to determine IC_50_ values. All experiments were performed in triplicate, and data are reported as mean ± SEM.

### 4.9 Primary Human PBMC Isolation and Culture

PBMCs from healthy donors were obtained from STEMCELL Technologies (Catalog# 70025) and cultured according to manufacturer recommendations. Cells were maintained in RPMI 1640 medium supplemented with 10% fetal bovine serum (FBS), L‐glutamine, and penicillin‐streptomycin (100 U/mL and 100 µg/mL, respectively) at 37°C in a humidified atmosphere containing 5% CO_2_. Cell viability following thawing was assessed using trypan blue exclusion before experiments.

### 4.10 T‐Cell Activation Assays in Primary PBMCs

PBMCs from healthy donors (*n* = 10 independent donors; STEMCELL Technologies, Catalog# 70025) were cultured in RPMI 1640 supplemented with 10% FBS and antibiotics. Cells were seeded at 2 × 10^5^ cells per well in 96‐well plates. T‐cell activation was induced using anti‐human CD3 (plate‐bound, 2 µg/mL) and soluble anti‐human CD28 (1 µg/mL). CIP‐3 was added at indicated concentrations (0.01–10 µm) at the time of stimulation. FR104 (anti‐CD28 monoclonal antibody, MedChemExpress, Catalog# HY‐P990587) (10 µg/mL) was included as a benchmark control. Supernatants were collected after 24 h, and IL‐2 and IFN‐γ concentrations were quantified using ELISA kits (STEMCELL Technologies, Catalog# 02006 and 02003). Each donor sample was tested in technical triplicate.

### 4.11 Agonist Activity Assessment

To evaluate intrinsic agonist potential, PBMCs were incubated with cyclic peptides in the absence of stimulation. Cytokine production was measured as described above and compared with baseline controls. Each donor sample was tested in technical triplicate.

### 4.12 Washout and Reversibility Experiments

To assess reversibility of CD28 inhibition, primary human PBMCs were preincubated with CIP‐3 (1 µm) or FR104 (anti‐CD28 monoclonal antibody, MedChemExpress, Catalog# HY‐P990587) (10 µg/mL) for 60 min at 37°C before stimulation. Following pretreatment, cells were washed three times with pre‐warmed RPMI 1640 medium (centrifugation at 400 × g for 5 min between washes) to remove unbound compound or antibody. Cells were then immediately restimulated with anti‐CD3/CD28 activation reagents (plate‐bound anti‐human CD3 antibody (clone OKT3, 2 µg/mL) together with soluble anti‐human CD28 antibody (1 µg/mL)) under the same conditions used in stimulation‐only controls. Parallel non‐washout conditions were included for comparison.

Cytokine production (IL‐2 and IFN‐γ) was measured in culture supernatants collected 24 h after restimulation using ELISA (STEMCELL Technologies, Catalog# 02006 and 02003). Functional recovery was calculated as a percentage of cytokine production relative to stimulated controls. Experiments were performed using PBMCs from independent donors (*n* = 10). Statistical comparisons were conducted using one‐way ANOVA with multiple comparisons. ELISA measurements were performed within the manufacturer‐reported dynamic range. Technical replicate variability was monitored, and intra‐assay coefficients of variation were <10% across measured samples.

### 4.13 Patient‐Derived PBMC Experiments

No written consent has been obtained from the patients, as there is no patient‐identifiable data included in this study. PBMCs from patients with ulcerative colitis (*n* = 5 independent donors; BioIVT, Catalog# HUMANPBMC‐0002203) were processed and cultured under identical conditions as healthy donor PBMCs. Cells were obtained from commercial vendors and were provided to investigators in a de‐identified manner. No identifiable donor information was available to investigators. Cells were stimulated with anti‐CD3/CD28 (plate‐bound anti‐human CD3 antibody (clone OKT3, 2 µg/mL) together with soluble anti‐human CD28 antibody (1 µg/mL)) in the presence of increasing concentrations of CIP‐3. Cytokine production was quantified by ELISA after 24 h. Statistical comparisons between peptide and antibody treatment were performed using paired two‐tailed *t*‐tests.

### 4.14 Murine CD28 Cross‐Reactivity Assessment

To evaluate functional cross‐reactivity of CIP‐3 with murine CD28, primary splenocytes were isolated from 8–10‐week‐old C57BL/6 mice. Spleens were mechanically dissociated through a 70‐µm cell strainer to obtain single‐cell suspensions. Red blood cells were lysed using ammonium‐chloride‐potassium (ACK) lysis buffer, and cells were washed twice with RPMI 1640 medium supplemented with 10% FBS, L‐glutamine, and penicillin‐streptomycin (100 U/mL and 100 µg/mL, respectively).

Splenocytes were seeded at 2 × 10^5^ cells per well in 96‐well plates and stimulated with plate‐bound anti‐mouse CD3 (2 µg/mL) and soluble anti‐mouse CD28 (1 µg/mL) antibodies. CIP‐3 was added at indicated concentrations (0.01–10 µm) at the time of stimulation. Cells were incubated at 37°C in 5% CO_2_ for 24 h.

Supernatants were collected and murine IL‐2 and IFN‐γ concentrations were quantified using ELISA kits (STEMCELL Technologies, Catalog# 02022 and 02020) according to the manufacturer's instructions. Experiments were performed using splenocytes from independent mice (*n* = 4 biological replicates), each measured in technical triplicate. Data were analyzed using one‐way ANOVA with appropriate post hoc testing.

### 4.15 Plasma Stability and Microsomal Stability

CIP‐3 stability in mouse plasma was evaluated by incubating the peptide (1 µm final concentration) in pooled mouse plasma at 37°C. Aliquots were collected at 0, 0.5, 1, 2, 4, and 6 h and quenched with ice‐cold acetonitrile containing an internal standard to precipitate plasma proteins. Samples were centrifuged at 15 000 × g for 10 min, and supernatants were analyzed by LC‐MS/MS. The percentage of remaining parent compound was calculated relative to time zero. Experiments were performed in triplicate.

Metabolic stability was assessed using pooled mouse and human liver microsomes (ThermoFisher Scientific, Catalog# MSMCPL and HMMCPL). CIP‐3 (1 µm) was incubated with microsomes (0.5 mg/mL protein) in potassium phosphate buffer (pH 7.4) at 37°C in the presence of an NADPH‐regenerating system. Reactions were initiated by the addition of NADPH and terminated at defined time points (0, 5, 15, 30, 45, and 60 min) by the addition of ice‐cold acetonitrile.

Samples were centrifuged, and supernatants were analyzed by LC‐MS/MS. The natural logarithm of the percentage remaining compound was plotted vs. time to determine the first‐order elimination rate constant (k). Intrinsic clearance (Cl_int_) and microsomal half‐life (t_1/2_) were calculated using standard equations. Experiments were performed in triplicate.

### 4.16 Animal Studies

Mice were housed and handled according to the guidelines approved by the Institutional Animal Care and Use Committee (IACUC) of our institution (protocol 2023‐0028).

### 4.17 PK Analysis of CIP‐3 in Mice

PK studies were conducted in male C57BL/6 mice (8–10 weeks old; 20–25 g). Animals were housed under standard conditions with ad libitum access to food and water. CIP‐3 was formulated in sterile PBS containing 5% DMSO and administered via subcutaneous (s.c.) injection at doses of 1 or 5 mg/kg (*n* = 4 per dose group). Blood samples (∼50–75 µL) were collected via submandibular venipuncture at the following time points post‐dose: 0.25, 0.5, 1, 2, 4, 8, 12, and 24 h. Blood was collected into EDTA‐coated tubes and centrifuged at 3,000 × g for 10 min at 4°C to obtain plasma. Plasma samples were stored at ‐80°C until analysis.

Plasma concentrations of CIP‐3 were quantified using a validated liquid chromatography‐tandem mass spectrometry (LC‐MS/MS) method. Briefly, plasma proteins were precipitated using acetonitrile containing an internal standard, followed by centrifugation and injection of the supernatant onto a reverse‐phase C18 analytical column. Chromatographic separation was achieved using a gradient of water and acetonitrile containing 0.1% formic acid. Detection was performed in positive electrospray ionization mode using multiple reaction monitoring (MRM). Calibration curves were generated using spiked plasma standards over a concentration range of 1–10 000 ng/mL. The lower limit of quantification (LLOQ) was 1 ng/mL. Quality control samples at low, medium, and high concentrations were included in each analytical run to ensure accuracy and precision.

PK parameters, including maximum plasma concentration (C_max_), time to maximum concentration (T_max_), area under the plasma concentration‐time curve from 0 to 24 h (AUC_0‐24 _h), terminal elimination half‐life (t_1/2_), and apparent clearance (CL/F), were calculated using non‐compartmental analysis in Phoenix WinNonlin (Certara). Terminal half‐life was determined from the slope of the log‐linear terminal phase. Data are reported as mean ± SD.

### 4.18 Acute In Vivo Tolerability Assessment

Healthy C57BL/6 mice (*n* = 5, 8–10 weeks old) received subcutaneous administration of CIP‐3 formulated in sterile PBS containing 5% DMSO at doses of 5 or 10 mg/kg. Vehicle‐treated mice received formulation buffer alone. Animals were monitored for clinical signs of distress, grooming behavior, mobility, and body weight changes throughout the study period.

For cytokine‐release assessment, blood samples were collected at defined time points following peptide administration, and serum concentrations of IL‐6, TNF‐α, and IFN‐γ were quantified using commercially available ELISA kits according to manufacturer instructions. At study termination, hematological and serum chemistry analyses were performed using whole blood and serum samples to evaluate potential systemic toxicity, including markers of hepatic and renal function.

### 4.19 Adoptive T Cell Transfer Model of Chronic Colitis

Chronic colitis was induced using the adoptive CD4^+^CD45RB^high^ T‐cell transfer model as previously described [42, 43, 44]. Briefly, spleens were harvested from 5‐week‐old BALB/c donor mice (Jackson Laboratory). Single‐cell suspensions were prepared by mechanical dissociation followed by filtration through a 70‐µm nylon mesh. CD4^+^ T cells were isolated using magnetic bead separation, and CD4^+^CD45RB^high^ T cells were purified by fluorescence‐activated cell sorting (FACS). Sorted cells were washed twice with sterile phosphate‐buffered saline (PBS) and resuspended at 1.5 × 10^6^ cells/mL in cold PBS.

Recipient C.B‐17 scid mice (7 weeks old; Taconic Biosciences) received 3 × 10^5^ CD4^+^CD45RB^high^ T cells via intravenous injection (200 µL per mouse). Mice were randomly assigned to treatment groups (*n* = 8 per group) following cell transfer. CIP‐3 was formulated in sterile PBS containing 5% DMSO and administered by daily subcutaneous injection at doses of 1 or 5 mg/kg. Vehicle‐treated mice received formulation buffer alone. No‐colitis control mice received daily vehicle injections (PBS containing 5% DMSO) according to the same dosing schedule but did not undergo T‐cell transfer. Treatment began on day 7 post‐transfer and continued for 30 days. Investigators were not blinded to treatment allocation. Disease progression was monitored longitudinally using a disease activity index (DAI) incorporating body weight loss, stool consistency, and fecal blood. Body weight was recorded three times per week. Body weight loss was scored as follows relative to initial body weight: 0 = <1% loss; 1 = 1%–5% loss; 2 = 5%–10% loss; 3 = 10%–15% loss; and 4 = >15% loss. Stool consistency was scored as follows: 0 = normal; 1 = soft but formed; 2 = loose; 3 = diarrhea. Fecal blood was assessed using a guaiac‐based test and scored as: 0 = negative; 1 = trace; 2 = positive; 3 = gross bleeding. The DAI score represents the combined average of these parameters.

At study termination (day 30), mice were euthanized, and colons were excised and measured from cecum to rectum under standardized tension. Serum was collected via cardiac puncture and centrifuged at 3000 × g for 10 min at 4°C. TNF‐α and IL‐6 concentrations were quantified using commercially available ELISA kits (ThermoFisher Scientific, Catalog# BMS607‐3 and KMC0061) according to the manufacturer's instructions.

### 4.20 Statistical Analysis

Data are presented as mean ± SEM unless otherwise indicated. Statistical analyses were performed using GraphPad Prism 10.4.1 software. Comparisons between groups were conducted using one‐way or two‐way analysis of variance (ANOVA) with appropriate post hoc tests as specified in figure legends. *P* values less than 0.05 were considered statistically significant.

## Author Contributions

The manuscript was written through the contributions of all authors. All authors have given approval to the final version of the manuscript. **Moustafa T. Gabr** and **Hongliang Duan** developed the conceptualization. **Katarzyna Kuncewicz**, **Saurabh Upadhyay**, and **Renjie Zhu** carried out the methodology, investigation, formal analysis, visualization, data curation, and validation. The original draft was written by **Katarzyna Kuncewicz**, **Saurabh Upadhyay**, **Renjie Zhu**, **Moustafa T. Gabr**, and **Hongliang Duan**, **Moustafa T. Gabr** and **Hongliang Duan** reviewed and edited the manuscript, acquired funding, and supervised the work. **Moustafa T. Gabr** managed the project.

## Conflicts of Interest

The authors declare no conflicts of interest.

## Supporting information




**Supporting File**: advs75892‐sup‐0001‐SuppMat.docx.

## Data Availability

The data that support the findings of this study are available from the corresponding author upon reasonable request.

## References

[1] D. M. Pardoll , “The Blockade of Immune Checkpoints in Cancer Immunotherapy,” Nature Reviews Cancer 12 (2012): 252–264, 10.1038/nrc3239. 22437870 PMC4856023

[2] K. Ma , Y. Xu , H. Cheng , K. Tang , J. Ma , and B. Huang , “T Cell‐Based Cancer Immunotherapy: Opportunities and Challenges,” Science Bulletin 70, no. 11 (2025): 1872–1890, 10.1016/j.scib.2025.03.054. 40221316

[3] S. C. Wei , C. R. Duffy , and J. P. Allison , “Fundamental Mechanisms of Immune Checkpoint Blockade Therapy,” Cancer Discovery 8 (2018): 1069–1086, 10.1158/2159-8290.CD-18-0367. 30115704

[4] F. S. Hodi , S. J. O'Day , D. F. McDermott , et al., “Improved Survival With Ipilimumab in Patients With Metastatic Melanoma,” New England Journal of Medicine 363 (2010): 711–723, 10.1056/NEJMoa1003466. 20525992 PMC3549297

[5] J. M. Mehnert , A. Varga , M. S. Brose , et al., “Safety and Antitumor Activity of the Anti–PD‐1 Antibody Pembrolizumab in Patients With Advanced, PD‐L1–Positive Papillary or Follicular Thyroid Cancer,” BMC Cancer 19 (2019): 196, 10.1186/s12885-019-5380-3. 30832606 PMC6399859

[6] A. Ribas and J. D. Wolchok , “Cancer Immunotherapy Using Checkpoint Blockade,” Science 359 (2018): 1350–1355, 10.1126/science.aar4060. 29567705 PMC7391259

[7] Q.‐Y. Su , J.‐T. Zhang , H.‐J. Gao , et al., “Mechanism and Clinical Utility of Abatacept in the Treatment of Rheumatoid Arthritis,” Expert Opinion on Drug Safety 25, no. 1 (2026): 59–70, 10.1080/14740338.2025.2505542. 40347194

[8] S. Upadhyay , B. Kaur , and M. T. Gabr , “CD28 and ICOS in Immune Regulation: Structural Insights and Therapeutic Targeting,” Bioorganic & Medicinal Chemistry Letters 127 (2025): 130310, 10.1016/j.bmcl.2025.130310. 40527414 PMC12213070

[9] R. Bai and W. Sun , “Crosstalk Between Tumor‐Associated Macrophages and the B7/CD28 Family in Immune Checkpoint Inhibitor‐Induced Immunotherapy,” Molecular and Cellular Biochemistry 481, no. 1 (2026): 127–137, 10.1007/s11010-025-05405-w. 41083896 PMC12906532

[10] S. H. Olejniczak , M. T. Lotze , and D. Skokos , “Second Signals for Cancer Immunotherapy,” Journal for ImmunoTherapy of Cancer 13, no. 9 (2025): 010530, 10.1136/jitc-2024-010530. PMC12458764 40992782

[11] Y. Lv , Y.‐L. Jin , Z. Zhou , et al., “The Interaction Between Dendritic Cells and T Follicular Helper Cells Drives Inflammatory Bowel Disease: A Review,” Frontiers in Immunology 17 (2026): 1725349, 10.3389/fimmu.2026.1725349. 41710888 PMC12909234

[12] K. Ma , W. Que , X. Hu , et al., “Combinations of Anti‐GITR Antibody and CD28 Superagonist Ameliorated Dextran Sodium Sulfate‐Induced Mouse Colitis,” Clinical and Experimental Immunology 208, no. 3 (2022): 340–350, 10.1093/cei/uxac039. 35511600 PMC9226153

[13] M. C. Genovese , J.‐C. Becker , M. Schiff , et al., “Abatacept for Rheumatoid Arthritis Refractory to Tumor Necrosis Factor α Inhibition,” New England Journal of Medicine 353 (2005): 1114–1123, 10.1056/NEJMoa050524. 16162882

[14] H. Kaplon and J. M. Reichert , “Antibodies to Watch in 2021,” mAbs 13 (2021): 1860476, 10.1080/19420862.2020.1860476. 33459118 PMC7833761

[15] B. Leader , Q. J. Baca , and D. E. Golan , “Protein Therapeutics: A Summary and Pharmacological Classification,” Nature Reviews Drug Discovery 7 (2008): 21–39, 10.1038/nrd2399. 18097458

[16] G. Walsh , “Biopharmaceutical Benchmarks 2018,” Nature Biotechnology 36 (2018): 1136–1145, 10.1038/nbt.4305. 30520869

[17] A. M. Scott , J. D. Wolchok , and L. J. Old , “Antibody Therapy of Cancer,” Nature Reviews Cancer 12 (2012): 278–287, 10.1038/nrc3236. 22437872

[18] A. L. Nelson , E. Dhimolea , and J. M. Reichert , “Development Trends for Human Monoclonal Antibody Therapeutics,” Nature Reviews Drug Discovery 9 (2010): 767–774, 10.1038/nrd3229. 20811384

[19] P. Chames , M. Van Regenmortel , E. Weiss , and D. Baty , “Therapeutic Antibodies: Successes, Limitations and Hopes for the Future,” British Journal of Pharmacology 157 (2009): 220–233, 10.1111/j.1476-5381.2009.00190.x. 19459844 PMC2697811

[20] G. Suntharalingam , M. R. Perry , S. Ward , et al., “Cytokine Storm in a Phase 1 Trial of the Anti‐CD28 Monoclonal Antibody TGN1412,” New England Journal of Medicine 355 (2006): 1018–1028, 10.1056/NEJMoa063842. 16908486

[21] T. Cierpicki and J. Grembecka , “Targeting Protein–Protein Interactions in Hematologic Malignancies,” Annual Review of Pathology: Mechanisms of Disease 20, no. 1 (2025): 275–301, 10.1146/annurev-pathmechdis-031521-033231. 39854187

[22] M. R. Arkin , Y. Tang , and J. A. Wells , “Small‐Molecule Inhibitors of Protein‐Protein Interactions: Progressing Toward the Reality,” Chemistry & Biology 21 (2014): 1102–1114, 10.1016/j.chembiol.2014.09.001. 25237857 PMC4179228

[23] W. R. Strohl , “Structure and Function of Therapeutic Antibodies Approved by the US FDA in 2024,” Antibody Therapeutics 8, no. 3 (2025): 197–237. 40994925 10.1093/abt/tbaf014 PMC12454936

[24] X. Ji , A. L. Nielsen , and C. Heinis , “Cyclic Peptides for Drug Development,” Angewandte Chemie International Edition 63, no. 3 (2024): 202308251, 10.1002/anie.202308251. 37870189

[25] P. C. Martian , M. Tertis , D. Leonte , N. Hadade , C. Cristea , and O. Crisan , “Cyclic Peptides: A Powerful Instrument for Advancing Biomedical Nanotechnologies and Drug Development,” Journal of Pharmaceutical and Biomedical Analysis 252 (2024): 116488, 10.1016/j.jpba.2024.116488. 39388867

[26] A. A. Vinogradov , Y. Yin , and H. Suga , “Macrocyclic Peptides as Drug Candidates: Recent Progress and Remaining Challenges,” Journal of the American Chemical Society 141 (2019): 4167–4181, 10.1021/jacs.8b13178. 30768253

[27] M. L. Merz , S. Habeshian , B. Li , et al., “De Novo Development of Small Cyclic Peptides That are Orally Bioavailable,” Nature Chemical Biology 20, no. 5 (2024): 624–633, 10.1038/s41589-023-01496-y. 38155304 PMC11062899

[28] S. You , G. McIntyre , and T. Passioura , “The Coming of Age of Cyclic Peptide Drugs: An Update on Discovery Technologies,” Expert Opinion on Drug Discovery 19, no. 8 (2024): 961–973, 10.1080/17460441.2024.2367024. 38872502

[29] H. Zhang and S. Chen , “Cyclic Peptide Drugs Approved in the Last Two Decades (2001–2021),” RSC Chemical Biology 3, no. 1: 18–31, 10.1039/D1CB00154J. PMC8729179 35128405

[30] R. Macarron , M. N. Banks , D. Bojanic , et al., “Impact of High‐Throughput Screening in Biomedical Research,” Nature Reviews Drug Discovery 10, no. 3 (2011): 188–195, 10.1038/nrd3368. 21358738

[31] D. K. Sell , B. Bakhshinejad , A. W. Sinkjaer , et al., “Using NGS to Uncover the Corruption of a Peptide Phage Display Selection,” Current Issues in Molecular Biology 46, no. 9 (2024): 10590–10605, 10.3390/cimb46090627. 39329979 PMC11431649

[32] S. A. Rettie , D. Juergens , V. Adebomi , et al., “Accurate De Novo Design of High‐Affinity Protein‐Binding Macrocycles Using Deep Learning,” Nature Chemical Biology 21 (2025): 1948–1956, 10.1038/s41589-025-01929-w. 40542165 PMC12643943

[33] C. W. Kosonocky , S. Alamdari , K. K. Yang , and A. P. Amini , “Closing the Loop: Experimentally Validated Methods in Artificial Intelligence–Driven Protein Design,” Current Opinion in Structural Biology 98 (2026): 103272, 10.1016/j.sbi.2026.103272. 42008922

[34] J. Jumper , R. Evans , A. Pritzel , et al., “Highly Accurate Protein Structure Prediction With AlphaFold,” Nature 596 (2021): 583–589, 10.1038/s41586-021-03819-2. 34265844 PMC8371605

[35] J. L. Watson , D. Juergens , N. R. Bennett , et al., “De Novo Design of Protein Structure and Function With RFdiffusion,” Nature 620 (2023): 1089–1100, 10.1038/s41586-023-06415-8. 37433327 PMC10468394

[36] C. Hsu , C. Fannjiang , and J. Listgarten , “Generative Models for Protein Structures and Sequences,” Nature Biotechnology 42 (2024): 196–199, 10.1038/s41587-023-02115-w. 38361069

[37] J. M. Stokes , K. Yang , K. Swanson , et al., “A Deep Learning Approach to Antibiotic Discovery,” Cell 180 (2020): 688–702.e13, 10.1016/j.cell.2020.01.021. 32084340 PMC8349178

[38] H. Lin , C. Zhu , T. Shang , et al., “HighPlay: Cyclic Peptide Sequence Design Based on Reinforcement Learning and Protein Structure Prediction,” Journal of Medicinal Chemistry 68, no. 11 (2025): 12047–12057, 10.1021/acs.jmedchem.5c00896. 40406858

[39] C. Zhang , C. Zhang , T. Shang , N. Zhu , X. Wu , and H. Duan , “HighFold: Accurately Predicting Structures of Cyclic Peptides and Complexes With Head‐To‐Tail and Disulfide Bridge Constraints,” Briefings in Bioinformatics 25, no. 3 (2024): bbae215, 10.1093/bib/bbae215. 38706323 PMC11070728

[40] P. Bryant , G. Pozzati , and A. Elofsson , “Improved Prediction of Protein‐Protein Interactions Using AlphaFold2,” Nature Communications 13, no. 1 (2022): 1265, 10.1038/s41467-022-28865-w. PMC8913741 35273146

[41] A. Harmalkar , S. Lyskov , and J. J. Gray , “Reliable Protein–Protein Docking With AlphaFold, Rosetta, and Replica Exchange,” Elife 13 (2025): RP94029, 10.7554/eLife.94029.3. 40424178 PMC12113263

[42] F. Powrie , M. W. Leach , S. Mauze , S. Menon , L. Barcomb Caddle , and R. L. Coffman , “Inhibition of Thl Responses Prevents Inflammatory Bowel Disease in Scid Mice Reconstituted With CD45RB^hi^ CD4^+^ T Cells,” Immunity 1, no. 7 (1994): 553–562, 10.1016/1074-7613(94)90045-0. 7600284

[43] P. J. Morrissey , K. Charrier , S. Braddy , D. Liggitt , and J. D. Watson , “CD4^+^ T Cells That Express High Levels of CD45RB Induce Wasting Disease When Transferred Into Congenic Severe Combined Immunodeficient Mice. Disease Development is Prevented by Cotransfer of Purified CD4^+^ T Cells,” The Journal of Experimental Medicine 178, no. 1 (1993): 237–244, 10.1084/jem.178.1.237. 8100269 PMC2191069

[44] F. Powrie , J. Carlino , M. W. Leach , S. Mauze , and R. L. Coffman , “A Critical Role for Transforming Growth Factor‐beta but Not Interleukin 4 in the Suppression of T Helper Type 1‐mediated Colitis by CD45RB(low) CD4^+^ T Cells,” The Journal of Experimental Medicine 183, no. 6 (1996): 2669–2674, 10.1084/jem.183.6.2669. 8676088 PMC2192626

