# Supplementary material for: AI‐Designed Cyclic Peptides Enable Controllable Modulation of the CD28 Immune Checkpoint
Source: Adv Sci (Weinh). 2026 May 30:e75892. Online ahead of print. doi: 10.1002/advs.75892 (PMC13336002; doi:10.1002/advs.75892)
Supplement: Supplementary file 1 — Supporting File: advs75892‐sup‐0001‐SuppMat.docx. [file ADVS-9999-e75892-s001.docx]

*Electronic Supplementary Information for*

**AI-designed cyclic peptides enable controllable modulation of the CD28 immune checkpoint**

AUTHOR NAMES

*Katarzyna Kuncewicz,^a,b#^ Saurabh Upadhyay,^a#^ Renjie Zhu,^c^* *[Hongliang Duan](https://www.sciencedirect.com/author/57213838871/hongliang-duan),^c^ Moustafa T. Gabr^a^**

AUTHOR ADDRESS

^a^Department of Radiology, Molecular Imaging Innovations Institute (MI3), Weill Cornell Medicine, New York, NY 10065, USA

^b^Department of Biomedical Chemistry, Faculty of Chemistry, University of Gdansk, Poland

^c^Faculty of Applied Sciences, Macao Polytechnic University, Macao 999078, China

* To whom correspondence should be addressed:

Moustafa T. Gabr ([mog4005@med.cornell.edu](mailto:mog4005@med.cornell.edu)).

^#^These authors contributed equally to this work

| **Contents** |  |
| --- | --- |
|  |  |
| Predicted binding mode of CIP-1 in complex with CD28  Predicted binding mode of CIP-2 in complex with CD28  Mass spectrum of CIP-1 | S3  S4  S5 |
| Mass spectrum of CIP-2 | S5 |
| Mass spectrum of CIP-3  Orthogonal validation of CIP-3 binding to CD28 by isothermal titration calorimetry (ITC) | S6  S7 |
| CIP-3 functionally inhibits murine CD28-mediated T-cell activation  Plasma concentration-time profile of CIP-3 following subcutaneous administration in C57BL/6 mice  Initial in vivo tolerability and cytokine-release assessment of CIP-3  HPLC trace of CIP-1  HPLC trace of CIP-2  HPLC trace of CIP-3 | S8  S9  S10  S11  S11  S11 |

**
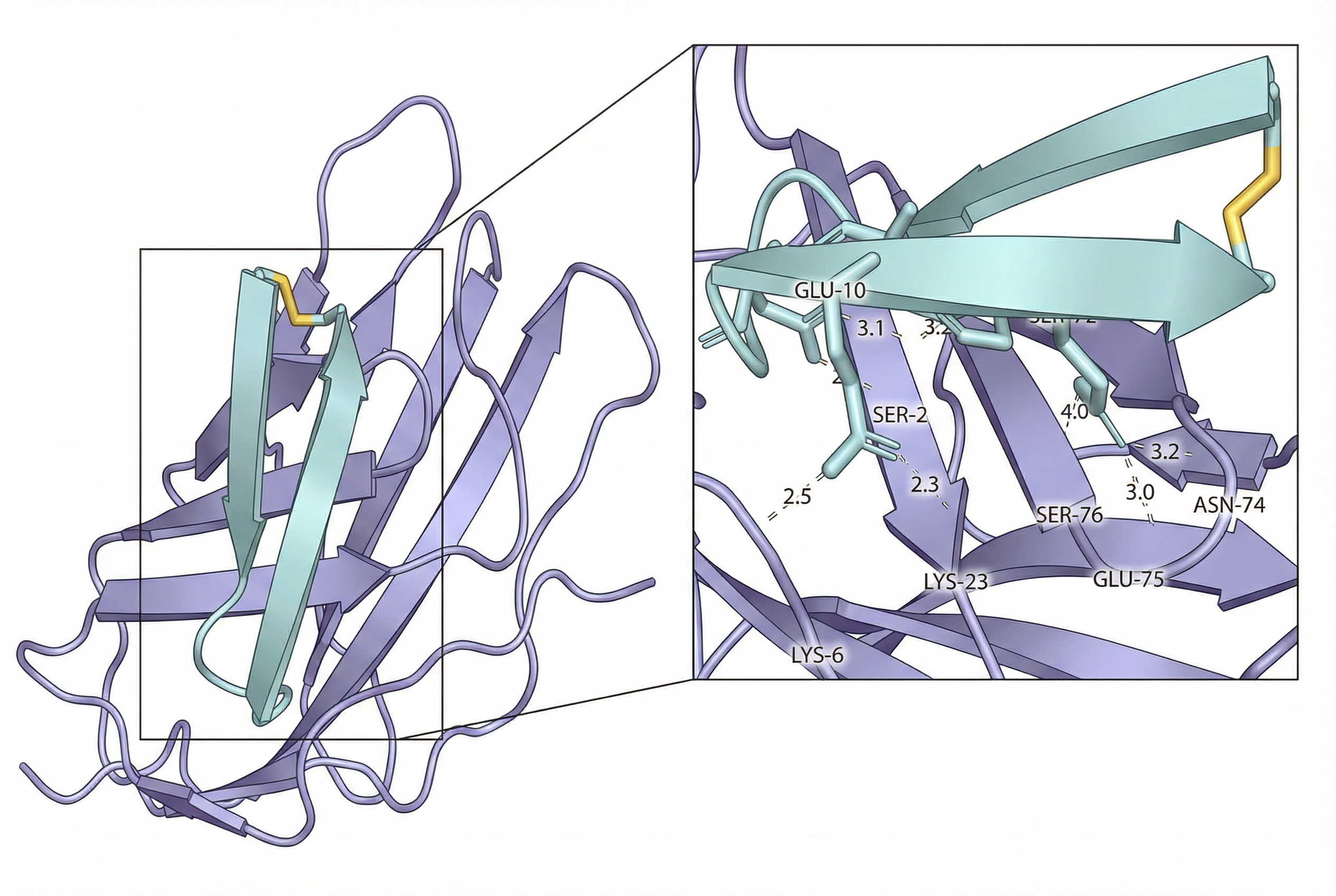
**

**Figure S1.** Predicted binding mode of CIP-1 in complex with CD28, with inset highlighting key intermolecular interactions and hydrogen bonds stabilizing the interface.


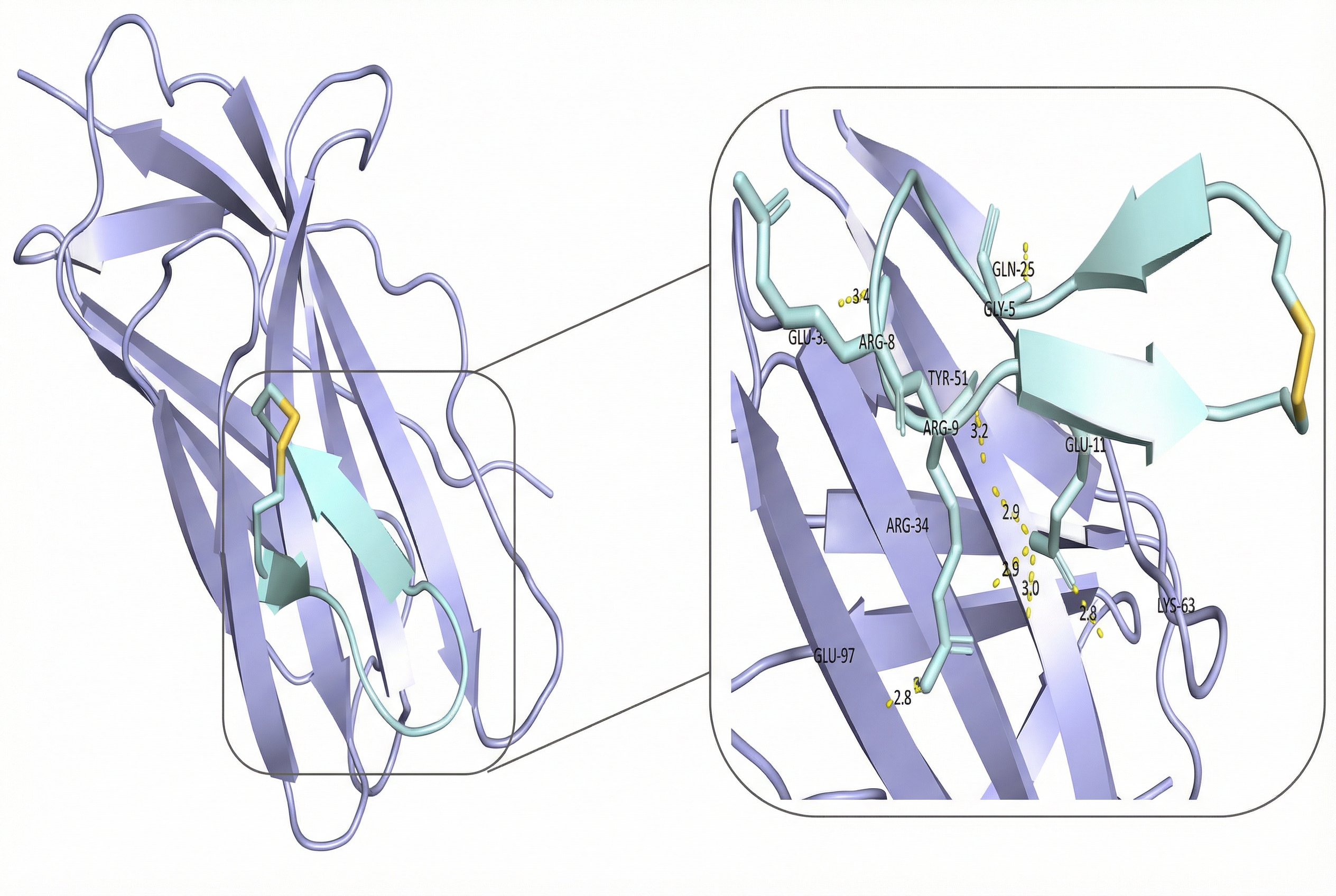


**Figure S2.** Predicted binding mode of CIP-2 in complex with CD28, with inset highlighting key intermolecular interactions and hydrogen bonds stabilizing the interface.


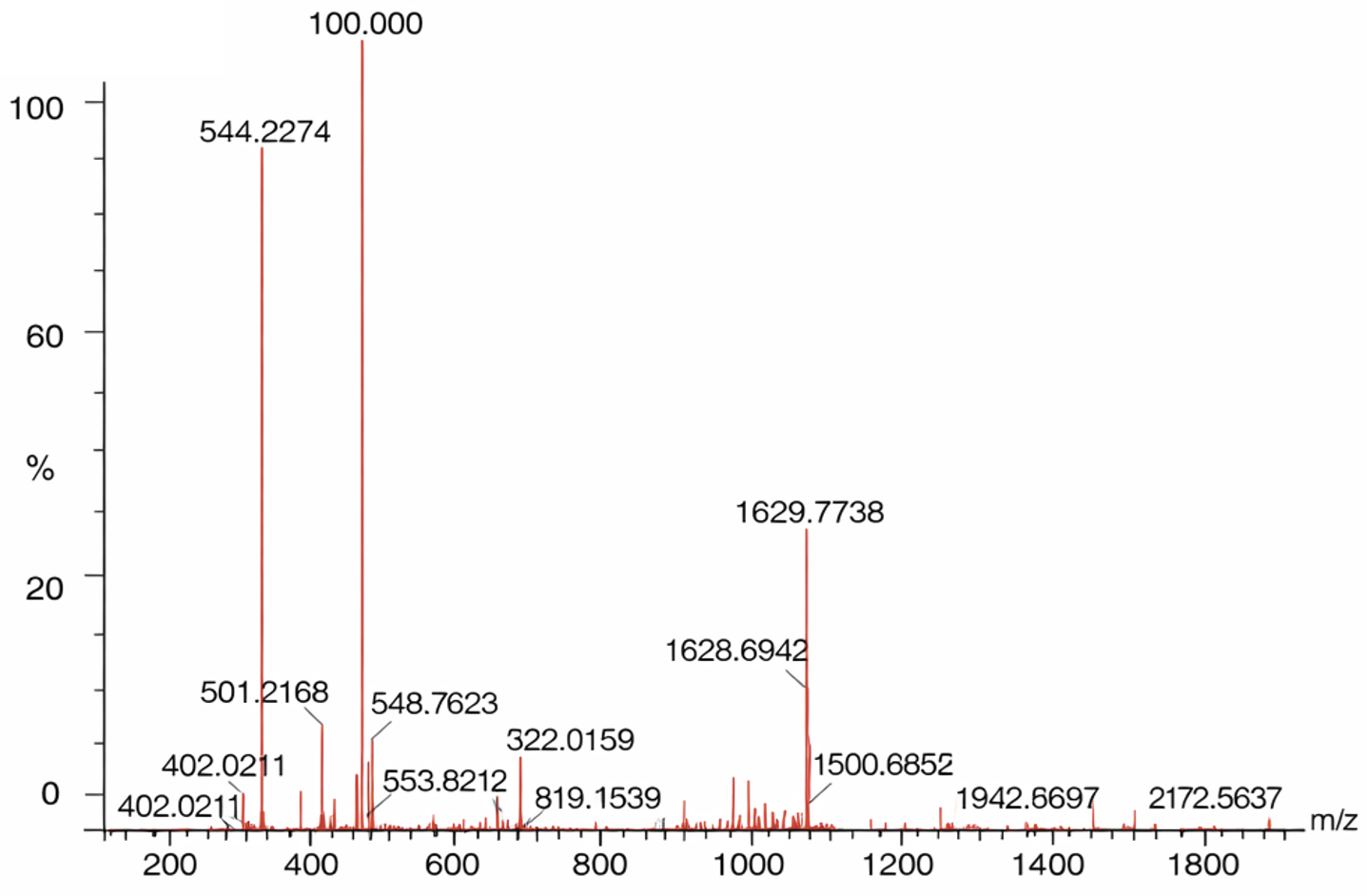


**Figure S3.** Mass spectrum of CIP-1.


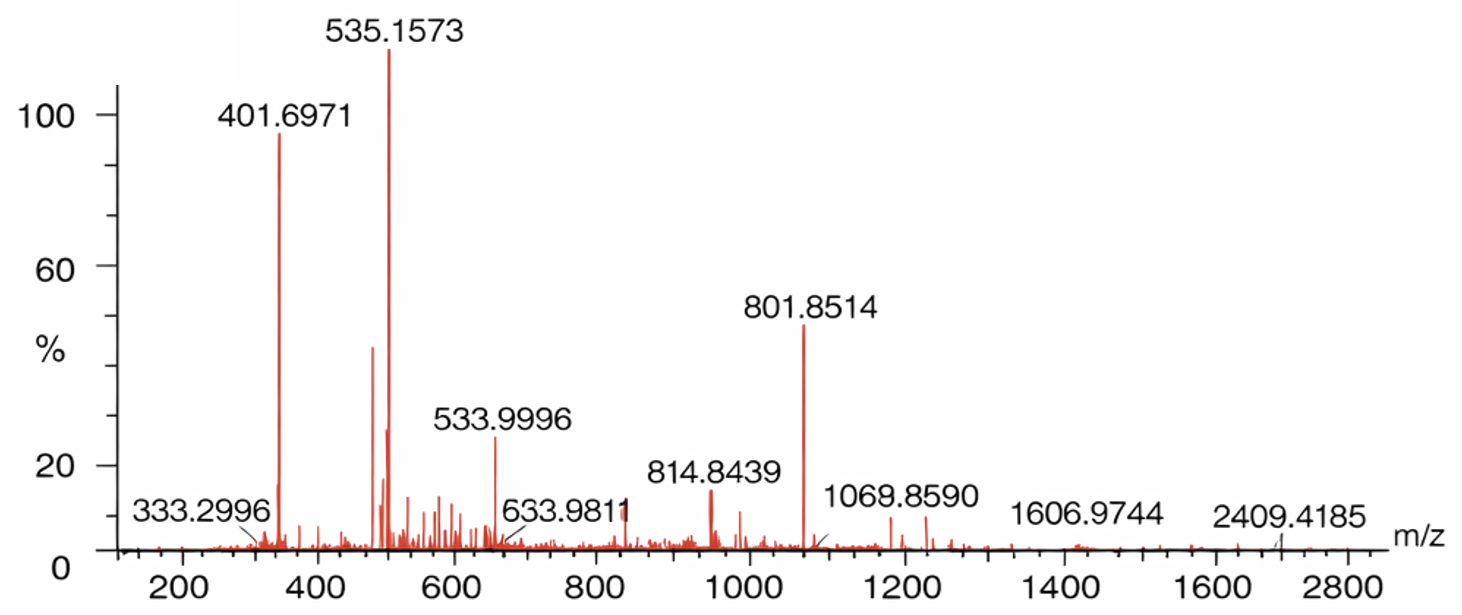


**Figure S4.** Mass spectrum of CIP-2.

**
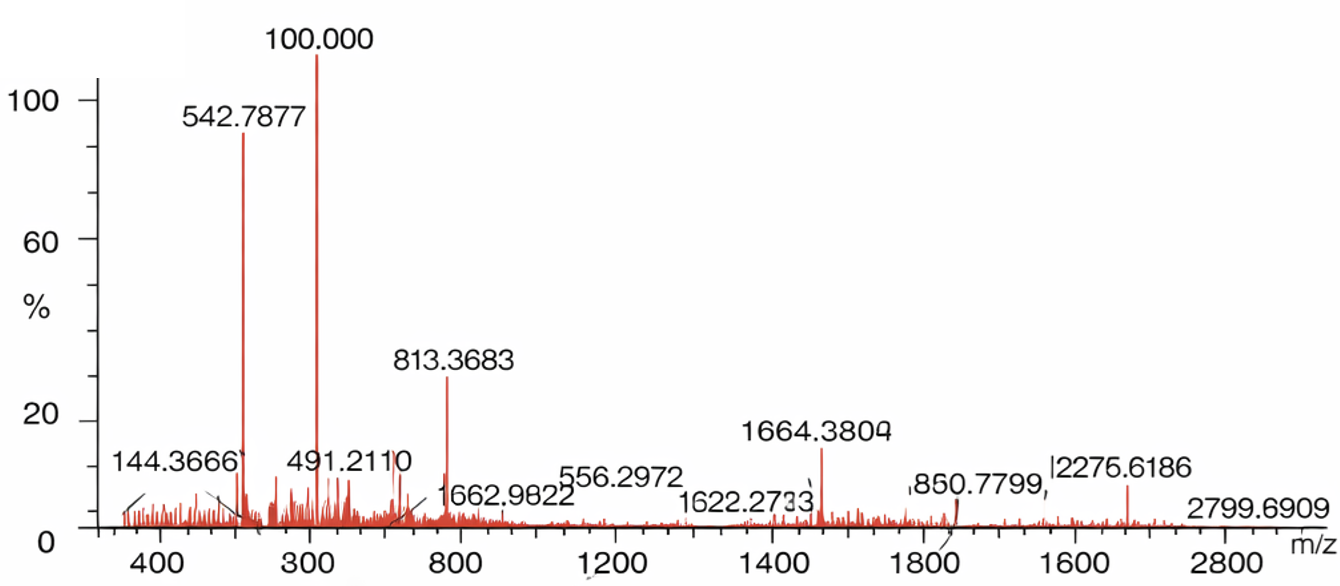
**

**Figure S5.** Mass spectrum of CIP-3.


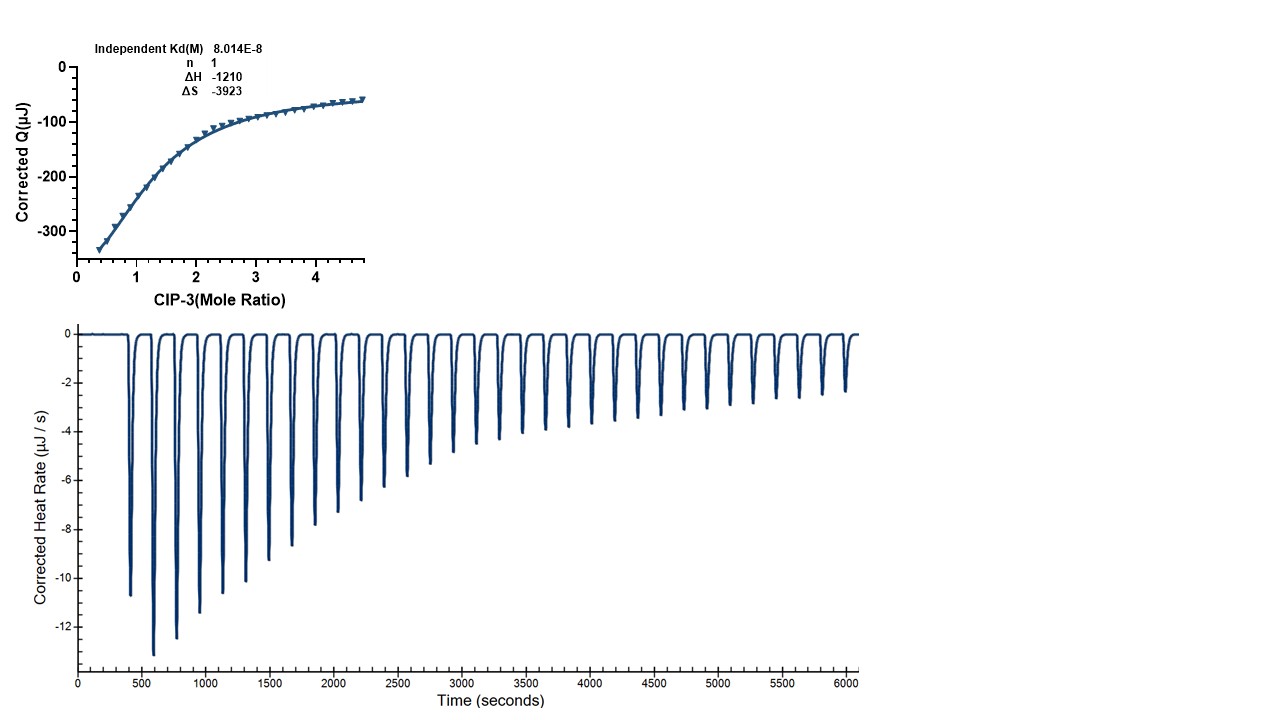


**Figure S6. Orthogonal validation of CIP-3 binding to CD28 by isothermal titration calorimetry (ITC).** (A) Representative ITC thermogram obtained by titration of CIP-3 into recombinant human CD28 extracellular domain (ECD) at 25 °C, showing progressive reduction in exothermic heat release with successive injections consistent with a saturable binding interaction. (B) Integrated binding isotherm fitted using a one-site binding model, demonstrating high-affinity interaction between CIP-3 and CD28 with an apparent dissociation constant (Kd) of approximately 80 nM and a binding stoichiometry consistent with a 1:1 interaction. Data support a specific interaction between CIP-3 and the CD28 extracellular domain and provide orthogonal biophysical validation of peptide binding.


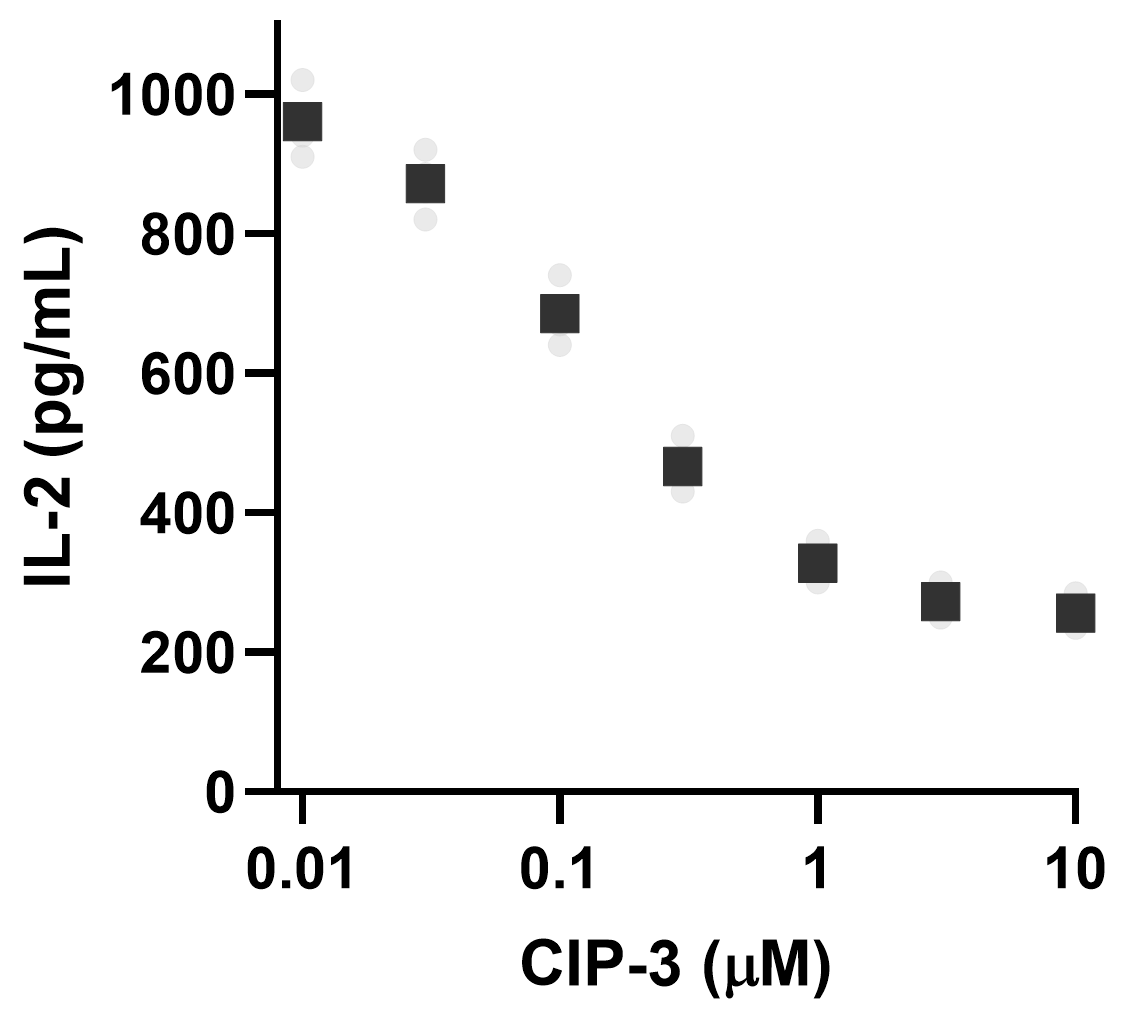


**Figure S7. CIP-3 functionally inhibits murine CD28-mediated T-cell activation.** Primary splenocytes isolated from C57BL/6 mice were stimulated with anti-CD3 and anti-CD28 antibodies in the presence of increasing concentrations of CIP-3 (0.01-10 µM). IL-2 production was quantified after 24 h by ELISA. CIP-3 suppressed murine CD28-dependent T-cell activation in a dose-dependent manner, with an apparent IC_50_ in the submicromolar range comparable to that observed in human PBMC assays. Anti-CD3 stimulation alone produced minimal IL-2 secretion, confirming CD28-dependent costimulatory signaling. Data represent mean ± SEM from independent spleen preparations (n = 3). Statistical analysis was performed using one-way ANOVA with multiple comparisons relative to stimulated control.


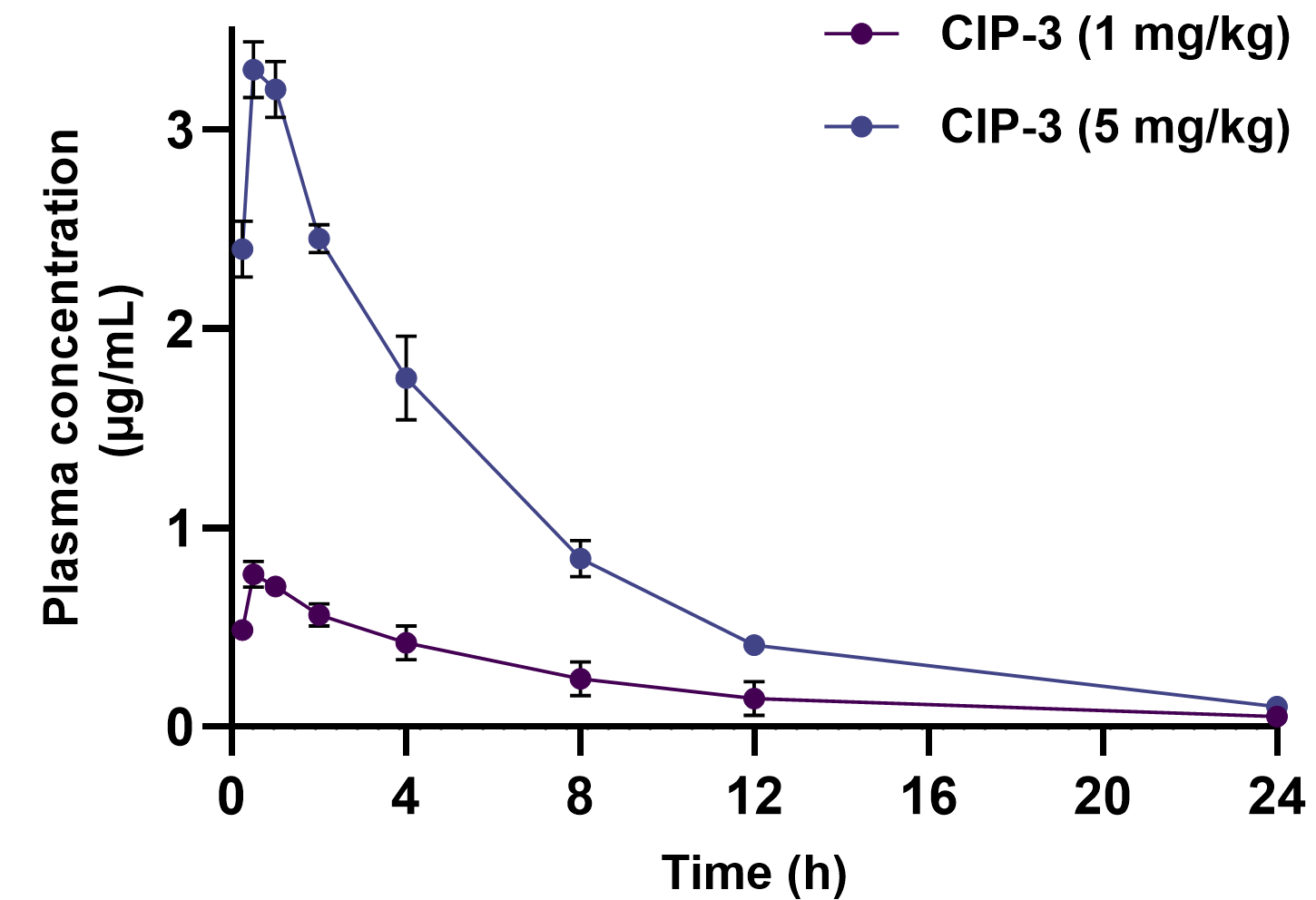


**Figure S8. Plasma concentration-time profile of CIP-3 following subcutaneous administration in C57BL/6 mice.** CIP-3 was administered as a single subcutaneous dose at 1 or 5 mg/kg, and plasma concentrations were quantified by LC-MS/MS over 24 h. Data are presented as mean ± SD (n = 4 mice per time point).


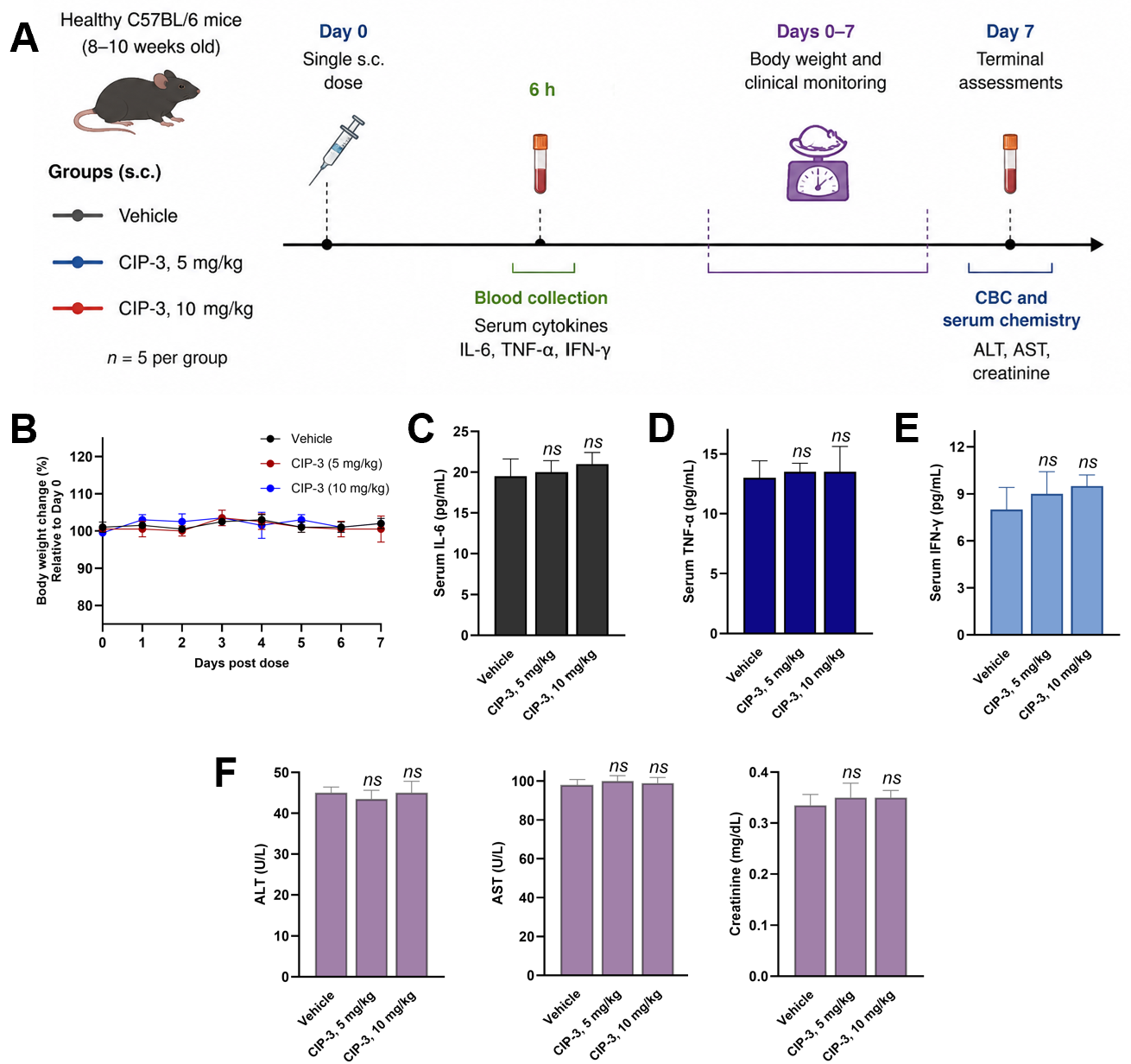


**Figure S9. Initial in vivo tolerability and cytokine-release assessment of CIP-3. (A)** Experimental schematic for acute tolerability evaluation in healthy C57BL/6 mice following subcutaneous administration of vehicle or CIP-3 at 5 or 10 mg/kg. Serum cytokines were measured 6 h post-dose, body weight and clinical appearance were monitored for 7 days, and hematology/serum chemistry analyses were performed at study termination. **(B)** Body weight monitoring over 7 days following a single subcutaneous dose of vehicle or CIP-3 demonstrated no treatment-associated weight loss. **(C-E)** Serum cytokine analysis performed 6 h after dosing revealed no detectable elevation in circulating IL-6 **(C)**, TNF-α **(D)**, or IFN-γ **(E)** levels following CIP-3 administration relative to vehicle-treated controls. **(F)** Serum chemistry analysis showed no significant alterations in markers of hepatic or renal function, including ALT, AST, and creatinine levels, following CIP-3 treatment. Data are presented as mean ± SEM (n = 5 mice per group). Statistical analysis was performed using one-way ANOVA with appropriate multiple-comparison testing.


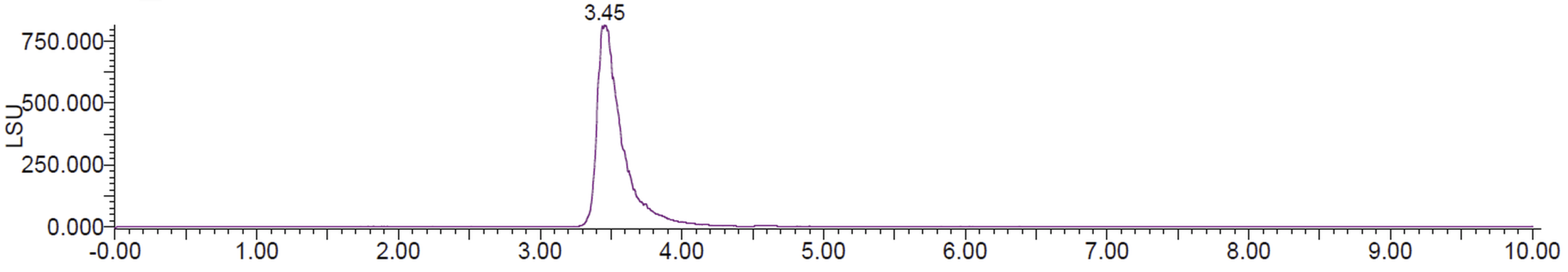


**Figure S10.** HPLC trace of CIP-1.


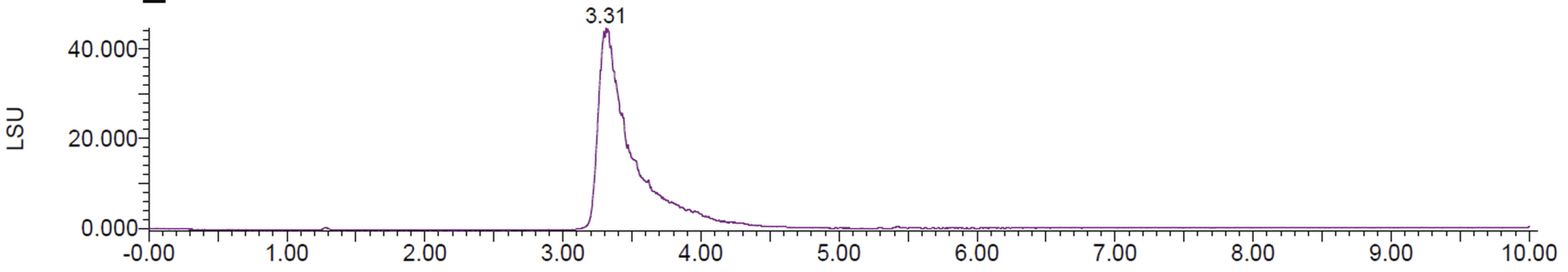


**Figure S11.** HPLC trace of CIP-2.


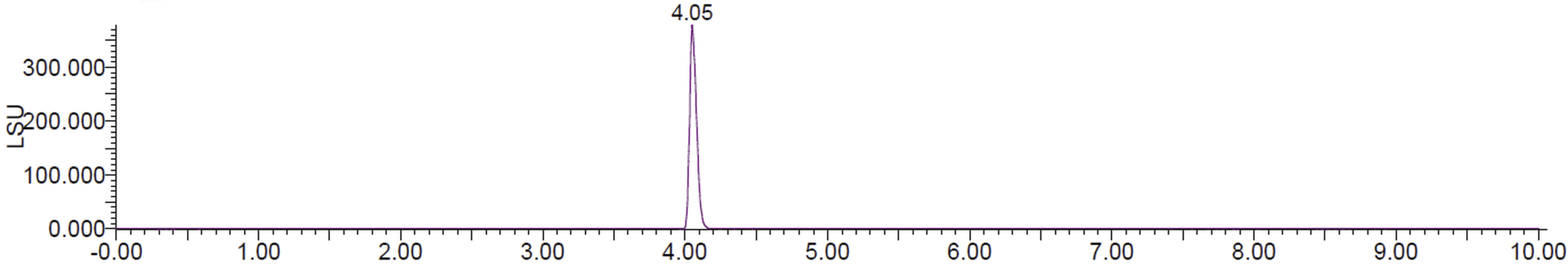


**Figure S12.** HPLC trace of CIP-3.
